# Multi-Population Selective Genotyping to Identify Soybean [*Glycine max* (L.) Merr.] Seed Protein and Oil QTLs

**DOI:** 10.1534/g3.116.027656

**Published:** 2016-04-01

**Authors:** Piyaporn Phansak, Watcharin Soonsuwon, David L. Hyten, Qijian Song, Perry B. Cregan, George L. Graef, James E. Specht

**Affiliations:** * Department of Agronomy and Horticulture, University of Nebraska, Lincoln, Nebraska 68583-0915; † Soybean Genomics and Improvement Laboratory, USDA-ARS, Beltsville, Maryland 20705-2325

**Keywords:** germplasm survey tool, QTLs: pleiotropy or linkage, rare alleles, nonunique SNP accessions, selection bias

## Abstract

Plant breeders continually generate ever-higher yielding cultivars, but also want to improve seed constituent value, which is mainly protein and oil, in soybean [*Glycine max* (L.) Merr.]. Identification of genetic loci governing those two traits would facilitate that effort. Though genome-wide association offers one such approach, selective genotyping of multiple biparental populations offers a complementary alternative, and was evaluated here, using 48 F_2:3_ populations (*n* = ∼224 plants) created by mating 48 high protein germplasm accessions to cultivars of similar maturity, but with normal seed protein content. All F_2:3_ progeny were phenotyped for seed protein and oil, but only 22 high and 22 low extreme progeny in each F_2:3_ phenotypic distribution were genotyped with a 1536-SNP chip (*ca*. 450 bimorphic SNPs detected per mating). A significant quantitative trait locus (QTL) on one or more chromosomes was detected for protein in 35 (73%), and for oil in 25 (52%), of the 48 matings, and these QTL exhibited additive effects of ≥ 4 g kg^–1^ and *R*^2^ values of 0.07 or more. These results demonstrated that a multiple-population selective genotyping strategy, when focused on matings between parental phenotype extremes, can be used successfully to identify germplasm accessions possessing large-effect QTL alleles. Such accessions would be of interest to breeders to serve as parental donors of those alleles in cultivar development programs, though 17 of the 48 accessions were not unique in terms of SNP genotype, indicating that diversity among high protein accessions in the germplasm collection is less than what might ordinarily be assumed.

Soybean [*Glycine max* (L.) Merr.], produced mainly in North and South America and Asia, is high in seed protein (40%) and oil (20%). These two seed constituents are consumed worldwide by domestic livestock, poultry, and fish (*i.e.*, soybean meal), and by humans (*i.e.*, cooking oil and Asian-style soybean food products). Soybean seed protein is inherited quantitatively, though more in an oligenic than a polygenic fashion, and is highly heritable (Burton 1987; Wehrmann *et al.* 1987; Wilcox 1998; Cober and Voldeng 2000). However, highly negative phenotypic and genotypic correlations of seed protein with seed yield and oil content have been routinely detected in biparental breeding populations (Burton 1987). Long-term selection for greater yield has also depressed protein and elevated oil (Rincker *et al.* 2014).

When soybean molecular markers became available in the 1990s (Keim *et al.* 1990), the detection and mapping of soybean quantitative trait loci (QTL) soon began. Diers *et al.* (1992) was the first to detect a major seed protein and oil QTL on soybean chromosome 20. Many seed protein and oil QTL have since been reported, and a listing of these, as well as QTL for other traits, can be found in SoyBase (Grant *et al.* 2010; http://www.soybase.org). However, nearly all of the protein and oil QTL reported to date have not been confirmed, and not one has yet been cloned. The additive effect values for these QTL are likely inflated due to the use of small population sizes in the published reports, because of an intrinsic QTL detection problem known as selection bias (Beavis 1998; Xu 2003; Broman and Sen 2009).

The parental sources of most high protein genes (*i.e.*, QTL alleles) used by soybean breeders are typically the high protein accessions acquired from the USDA Soybean Germplasm Collection. Trait data are documented in the Germplasm Resources Information Network (GRIN) for the 21,728 *G. max* accessions present in the collection as of December 31, 2015 (http://ars-grin.gov/npgs). For just the 12,141 *G. max* accessions in maturity groups (MGs) 0–IV, substantive variation clearly exists for each trait (Supplemental Material, Figure S1), though it is also evident in this large set of accessions that seed protein exhibits a negative relationship with seed oil and yield. Knowing the allelic status of the seed protein QTL in these accessions would help breeders select donor parents, and also allow a focus on those QTL that have an allele that exerts a large positive additive effect on seed protein, coupled with a smaller negative pleiotropic effect on seed oil and yield.

The allelic diversity of soybean seed protein QTL in germplasm collections would seem to be best addressed using an association analysis method (Thornsberry *et al.* 2001; Semagn *et al.* 2010; Korte and Farlow 2013). Bandillo *et al.* (2015) recently conducted a genome-wide association study (GWAS) involving all of the *G. max* accessions in the collection with protein and oil phenotypic data (*i.e.*, *n* = 12,116) that had been genotyped with a 50K single nucleotide polymorphism (SNP) chip. Strong signals were detected on chromosomes 20 and 15, plus weaker signals on chromosomes 13, 6, and 5. The authors of the latter study noted that the use of large numbers of accessions for GWAS greatly improved statistical power, and provided exceptional map resolution for the ultimate identification and cloning of the causal genes underpinning the major QTL on chromosomes 20 and 15. However, the rarity of a high protein allele at a QTL (and the coincident rarity of linkage-coupled alleles at QTL-flanking SNPs) can be an issue when selecting accession samples for GWAS. This was evident when Bandillo *et al.* (2015) stratified the 12K accessions into smaller subsets by sorting them into seven different countries of origin, or alternatively, into eight different MG classes. In some country subsets, and some MG subsets, the frequency of the protein-enhancing alleles of the QTL was lower than the GWAS minimum allele frequency (MAF) cutoff value, thereby resulting in no detection of one or two or all of the above-listed QTL. Thus, despite the power offered by GWAS in QTL detection, this “rare allele” problem can result in QTL not being detected in GWAS that were previously identified and confirmed to be present in biparental QTL mapping populations—wherein the frequency of the two parental alleles at any *segregating* high protein QTL is always expected to be near 0.5.

Selective genotyping (SG) was a term first used by Lander and Botstein (1989) to describe those cases of QTL mapping in which only the most informative individuals—those occupying the lowest and highest tails of a phenotyped trait distribution—were genotyped. A trait-based QTL detection approach had previously been conducted in plants (Stuber *et al.* 1980, 1982). Lebowitz *et al.* (1987) and Darvasi and Soller (1992) subsequently formulated and discussed the statistical issues relevant to SG. When using SG, one must still phenotype the entire population to conduct an unbiased QTL analysis (Darvasi 1997; Darvasi and Soller 1992; Muranty and Goffinet 1997; Sen *et al.* 2005, 2009). Optimal efficiency is usually achieved with SG if one does not genotype more than the upper (and lower) 20–25% of the mapping population for a given trait. Sun *et al.* (2010) noted that the optimum size of the tail proportion of a population was governed by a balance between QTL detection power and total cost, which was reflective of the ratio between genotyping and phenotyping costs.

Soybean breeders typically rely on near-infrared reflection (NIR) instrumentation to estimate the seed protein and oil content of germplasm lines (Hymowitz *et al.* 1974). About 100–200 soybean seed samples can be nondestructively phenotyped per hour of effort. Seed protein and oil phenotyping is relatively inexpensive, though it is labor-intensive. Thus, SG would seem to offer a cost-effective means of conducting a QTL analysis of multiple biparental mapping populations segregating for major-effect high and low alleles at seed protein QTL.

Ayoub and Mather (2002) demonstrated that if SG had been applied to just the lowest 10% and highest 10% of each trait in a North American barley mapping population, the resultant SG-based QTL analyses would have been sufficient to detect *all* of the grain and malt quality QTL that had been identified based on a genotyping of the entire population of about 140–150 lines. This publication triggered our interest in using a multiple mapping population SG approach as a means of surveying a large sample of high protein soybean germplasm accessions for the presence of high protein alleles at known and unknown QTL. The availability of a 1536-SNP marker assay—the Universal Soybean Linkage Panel 1.0 (USLP 1.0) developed by Hyten *et al.* (2010)—in a 96-well genotypic sample format, was another contributing factor leading us to examine the utility of a multi-population SG strategy to identify alleles of QTL that condition high seed protein, but which may have a low frequency in germplasm collection accessions. Rare QTL alleles are difficult to detect in a GWAS, both in theory (Raychaudhuri 2011; Ladouceur *et al.* 2012), and in practice (Bandillo *et al.* 2015). In that regard, we hypothesized that a SG strategy might mitigate the traditional GWAS rare-allele problem (Korte and Farlow 2013).

We thus report here on the use of a SG-based QTL analysis to survey 48 soybean populations, averaging about 224 F_2_ plants, derived from the mating of 48 high-seed-protein soybean germplasm accessions in seven MGs (spanning 000 to IV) to one of seven high-yielding lower protein cultivars with a matching MG. The ultimate objective of this study was to discern whether a multiple-population SG approach could be used to identify and map both known and unknown protein QTL in these high protein accessions that might serve as donor parents.

## Materials and Methods

### Parents and population development

To minimize the segregation of major genes controlling date of flowering/maturity in the F_2_ generation, the high seed protein accessions of a given MG were mated to a high-yield cultivar of ordinary seed protein content of the same MG. The parents are shown in Table 1, with each M-code-designated male parent listed just below the respective set of female parents to which that male parent was mated (except for MG V P1183, which was reciprocally mated to MG IV P1181M). The phenotypic data in Table 1 (except as footnoted) were extracted from the GRIN website (https://npgsweb.ars-grin.gov/gringlobal/descriptors.aspx). The 48 female parents had a GRIN-based seed protein content that ranged from 473 to 529 g kg^–1^ (*i.e.*, zero seed moisture, dry weight basis), whereas the range for the seven male parents was 382 to 430 g kg^–1^ (Table 1); the latter range is typical for cultivars currently being grown in the North Central United States soybean production area.

**Table 1:** The 48 high seed protein accession female parents, and the seven ordinary seed protein cultivar male parents (M-suffixed codes), ordered by soybean maturity group (MG), and then by mating and parent code. The seed protein and oil values listed for the female and male parents are those available in the Germplasm Resources Information Network (GRIN) website (but see footnote for exceptions).

Mating No.	Parent Code*^a^*	Maturity Group	Seed*^b^*		Germplasm Accession			Stem Habit	Flower Color	Pubescence		Pod Color	Seed Coat		Hilum Color
			Protein	Oil	Number*^c^*	Name (if Any)	Origin*^d^*			Color	Form		Luster	Color	
			g kg^–1^					Descriptor Code*^e^*							
1	P1001	000	529	151	PI 153296	V-4	Belgium	D	P	T	E	Br	S	Gn	Bl
2	P1002	000	504	158	PI 189963	Geant Vert	France	D	P	T	E	Br	D	Gn	Bl
3	P1003	000	522	155	PI 548399	Pando	South Korea	D	P	T	E	Br	S	Gn	Bl
4	P1004	000	477	156	PI 372423	Ronset 4	France	D	P	T	E	Br	I	Lgn	Bl
5	P1005	000	512	157	FC 30687	Kosodiguri Ext Early	Japan	D	P	T	E	Br	I	Gn	Bl
6	P1006	000	511	158	PI 153293	N-34	Belgium	D	P	T	E	Br	S	Gn	Bl
7	P1007	000	478	161	PI 372412	Hercumft	Germany	D	P	T	E	Tn	S	Lgn	Bl
8	P1009	000	522	159	PI 548414	Sioux	Japan	D	P	T	E	Br	S	Gn	Bl
—	P1021M	000	430	199	PI 567787	OAC Vision	Canada	N	P	T	E	Br	D	Y	Tn
9	P1022	00	507	158	PI 153302	V-16	Belgium	D	P	T	E	Br	S	Gn	Bl
10	P1023	00	526	157	PI 159764	—	South Korea	D	P	T	E	Br	S	Gn	Bl
11	P1024	00	485	164	PI 438415	Ronest 4	France	S	P	T	E	Br	I	Gn	Bl
12	P1025	00	508	147	PI 153301	V-14	Belgium	D	P	T	E	Br	S	Gn	Bl
13	P1026	00	489	173	PI 189880	Bitterhof	Germany	N	P	G	E	Br	S	Y	Y
14	P1027	00	510	148	PI 153297	V-6	Belgium	D	P	T	E	Br	S	Gn	Bl
15	P2211	00	(-)	(-)	—	HHP	Illinois (?)	N	Lp	G	?	Br	?	Ib*^f^*	Ib
16	P2212	00	(486)	(164)	—	AC *Proteus*	Canada	N	P	T	?	Br	D	Y	Br
17	P2213	00	(456)	(181)	—	AC Proteina	Canada	N	P	T	?	Br	?	Y	Br
—	P1038M	00	415	185	PI 602897	Jim	North Dakota	N	P	G	E	Br	I	Y	Y
18	P1039	0	480	144	PI 427138	Choseng No. 1	South Korea	D	W	G	A	Br	D	Y	Bf
19	P1040	0	488	195	PI 261469	Wasedaizu No. 1	Japan	N	W	G	A	Br	D	Y	Bf
20	P1041	0	485	177	PI 181571	No. 58	Japan	N	W	G	A	Br	D	Y	Bf
21	P1042	0	483	150	PI 424148	Shirome	South Korea	N	W	G	A	Br	I	Y	Bf
22	P1043	0	473	156	PI 423954	Shirome	Japan	D	W	G	Sa	Br	D	Y	Bf
23	P1044	0	494	160	PI 154196	No. 51	Netherlands	D	P	T	E	Br	D	Gn	Bl
—	P1053M	0	403	196	PI 602594	MN0301	Minnesota	N	P	G	E	Br	I	Y	Y
24	P1054	I	484	155	PI 437088A	DV-147	Russian Federation (Far East)	N	P	T	E	Br	D	Y	Br
25	P1055	I	514	144	PI 423949	Saikai 20	Japan	D	Lp	G	A	Br	I	Y	Bf
26	P1056	I	495	141	PI 427141	Seuhae No. 20	South Korea	S	P	T	E	Br	D	Y	Br
27	P1057	I	482	138	PI 437716A	Sjuj-dja-pyn-da-do	China	S	P	G	Sa	Br	I	Y	Bf
28	P1058	I	489	149	PI 423942	Saikai 1	Japan	D	P	G	A	Tn	I	Y	Bf
—	P1074M	I	(407)	(195)	PI 602593	MN1301	Minnesota	N	W	G	E	Br	D	Y	Y
29	P1075	II	499	157	PI 423948A	Saikai 18	Japan	N	B	G	E	Br	S	Y	Bf
30	P1076	II	482	154	PI 437112A	VIR 249	Russian Federation (Far East)	N	W	G	E	Tn	S	Y	Y
31	P1098	II	484	191	PI 548608	Provar	Iowa	N	P	T	E	Br	D	Y	Br
—	P1106M	II	382	195	PI 597386	Dwight	Illinois	N	P	T	E	Tn	D	Y	Bl
32	P1107	III	504	132	PI 445845	Szu yueh pa	China	D	W	G	A	Tn	D	Y	Bf
33	P1108	III	494	167	PI 398516	KAERI-GNT 310-1	South Korea	D	P	Lt	E	Br	D	Y	Y
34	P1109	III	477	170	PI 91725-4	Akazu	South Korea	D	W	G	Sa	Br	D	Y	Bf
35	P1110	III	493	165	PI 340011	—	South Korea	D	P	G	E	Br	D	Y	Y
36	P1111	III	478	162	PI 243532	Kariho-takiya	Japan	D	W	T	E	Dbr	S	Y	Br
37	P1113	III	497	168	PI 408138C	KAS 640-7	South Korea	D	P	G	E	Br	D	Y	Y
38	P1121	III	494	177	PI 398672	KAERI-GNT 301-1	South Korea	D	Dp	T	E	Br	S	Rbr*^f^*	Rbr
39	P1122	III	484	184	PI 360843	Oshimashirome	Japan	N	W	G	E	Br	I	Y	Y
—	P1137M	III	411	194	PI 597387	Pana	Illinois	N	P	G	E	Br	D	Y	Bf
40	P1138	IV	479	157	PI 253666A	No. 17	China	N	W	G	Sa	Br	I	Y	Bf
41	P1139	IV	507	151	PI 407788A	ORD 8113	South Korea	D	P	G	E	Tn	S	Y	Bf
42	P1140	IV	493	155	PI 424286	KAS 239-4	South Korea	D	P	G	E	Tn	D	Y	Bf
43	P1142	IV	488	166	PI 407877B	KAERI 511-11	South Korea	D	P	G	E	Br	D	Y	Bf
44	P1143	IV	488	158	PI 398704	KAS 330-9-1	South Korea	D	P	G	E	Br	I	Y	Bf
45	P1145	IV	491	160	PI 398970	KLS 630-1	South Korea	D	P	G	E	Tn	D	Y	Lbf
46	P1146	IV	493	159	PI 407823	—	South Korea	D	P	G	E	Tn	I	Y	Bf
47	P1152	IV	492	161	PI 407773B	KAS 330-9-2	South Korea	D	W	T	E	Tn	I	Y	Bl
—	P1181M	IV	424	180	PI 606748	Rend	Illinois	N	W	G	E	Br	D	Y	Bf
48	P1183	V	476	195	PI 458256	KAS 578-1	South Korea	D	P	G	Sa	Br	I	Y	Y

The seed protein and oil values listed for the female and male parents are those available in the Germplasm Resources Information Network (GRIN) website (but see footnote for exceptions).

a Nebraska field nursery parent identification number. The suffix M denotes a male parent (*i.e.*, the seven agronomic cultivars mated to females of the same MG).

b Seed protein and oil values are not available for these four non-GRIN entries: HHP—Brummer *et al.* (1997) provided details on this high protein accession and its likely source; AC *Proteus* and AC Proteina—protein and oil values shown here were reported by Voldeng *et al.* (1996); MN1301—protein and oil values were reported in Hill *et al.* (2008).

c The solid-line and dashed-lined underscoring identifies two groups of accessions that, within each group, were not unique in terms of their SNP genotype.

d The non-Asian origin listed for many high protein accessions is, in fact, simply the location of the organization (*i.e.*, mostly European germplasm collection agencies) that donated those accessions to the USDA germplasm collection, but did not provide information as to where in Asia the accession was originally collected.

e GRIN descriptor codes: D, determinate; IN, indeterminate; S, semi-determinate; Dp, dark purple; Lp, light purple; B, blue; W, white; Bl, black; Ib, imperfect black; Y, yellow; Br, brown; Bf, buff; Tn, tan; T, tawny; G, gray; Gn, green; Lgn, light green; E, erect; A, appressed; Sa, semi-appressed; Rb, red-brown; S, shiny; I, intermediate; D, dull.

f A nonyellow darkly pigmented seed coat color interferes with NIR-based protein and oil measurements. With respect to these two specific female parents, we discarded the homozygous recessive fraction (1/4) of the total F_2_ plants that produced F_2:3_ seed progenies that had darkly pigmented (nonyellow) colored seed coats.

Pollinations for all 48 matings were made in the summer growing season, and were successful in terms of generating putative F_1_ seeds that were individually hand-harvested in the fall and packaged by pod. The F_1_ to F_2_ generation advance was conducted in a greenhouse. To ensure the authenticity of putative F_1_ plants, a known parentally polymorphic SSR marker was used to genotype each F_1_ to confirm F_1_ hybridity in the 48 matings. Marker-confirmed F_1_ plants from each mating were individually harvested at maturity to obtain F_2_ seeds. Population-specific F_1:2_ seed progeny were planted the following summer into 48 single rows (30 m long; 76.2 cm row spacing). About 300 F_2_ seeds of each mating were planted in a row, with a goal of obtaining about 250 F_2_ plants bearing F_3_ seed. Parental seed and confirmed F_1_ seed also were planted in repetitive sections of the same row. All F_2_ plants were numerically tagged after emergence (during leaf tissue collection), and surviving tagged plants were gathered at maturity to be individually threshed to obtain F_3_ seed.

### Phenotypic trait measurement

The F_2:3_ seed progenies, the F_1:2_ seed progenies, and parental seed of a given mating, plus seed of four checks (*i.e.*, breeding lines known to be low or high in seed protein), were evaluated for seed protein, oil, and moisture content using a near-infrared reflectance (NIR) analyzer (Infratec model 1255 NIR Food and Feed Grain Analyzer, Ultra Tec Manufacturing Inc. Santa Ana, CA). The four check samples were used at the beginning and end of each day to confirm that the NIR instrument was operating during the day within its performance standards. Seed protein and oil values were output on a zero per cent seed moisture basis.

One complete replicate of the NIR-measured protein (and oil) data was obtained for all available F_2:3_ progenies in each of the 48 populations. Though each population required about 2 hr of assay time, only two (and on occasion, three) 2-hr assays could be conducted on a given day due to worker availability, instrument warm-up and prep time, etc. Thus, this 48-population NIR assay effort required about five contiguous weeks of workdays to complete. The F_2:3_ seed progeny in each mating were then ranked from lowest to highest based on their measured seed protein value. After completing a second replicate of NIR-assays of all progenies in just two populations (*i.e.*, matings 43 and 44; Table 1), it was determined that the F_2:3_ seed progenies present in highest and lowest 10% fractions of the first and second replicate assays were essentially the same progenies (data not shown). Thus, to reduce the phenotyping effort and time required to identify the F_2:3_ progenies occupying just the lowest and highest 10% fractions, a second replicate of NIR measurement was performed only on the highest and lowest 20% fractions in each of the remaining 46 populations. In each low and high 20% of 2-rep means, those F_2:3_ seed progenies ranking at the extreme ends of those 20% fractions were selected to become the corresponding 10% tail fractions of the seed protein distribution. Leaf tissue samples of the F_2_ plant progenitors of just these extreme progenies (*i.e.*, 22 high and 22 low protein) were subsequently used for SG.

### SNP marker genotyping

Standard methods for leaf collection and DNA extraction methods were used (for details, see File S1). All steps in the SNP genotyping assays of the parental, F_1_, and F_2_ DNA samples of the 48 SG populations (*i.e.*, a total of 24 plates) were conducted by personnel at the Soybean Genomics and Improvement Laboratory, USDA-ARS, BARC-West, Beltsville, MD, using the Illumina GoldenGate assay and an Illumina Beadstation 500 (Illumina Inc., San Diego, CA). A soybean-specific USLP 1.0 GoldenGate assay had been developed by Hyten *et al.* (2010) for 1536 SNP markers that were distributed (relatively) uniformly across the 20 chromosomes of the soybean genome. Automatic genotype calling for each SNP locus in each DNA sample in the first 10 two-population plates was conducted using Illumina GeneCall software, but the newer BeadStudio software was used for the 14 remaining two-population plates. All automated genotype call output was manually examined and adjusted as needed. Illumina base-pair allele calls were phase-translated into two-character genotype codes of **AA** for the high yield (normal protein) elite male parent, **BB** for the high protein accession female parent, and **AB** for the F_1_ progenitor of the F_2_ population, but were subsequently converted to single character codes of **A H B –** (*i.e.*, dash was assigned to missing genotypes) for use with linkage and analysis software.

### Phenotypic data analysis

The distributional statistics of the F_2:3_ phenotypic data collected for seed protein and oil content, and their phenotypic correlation (in each population), were examined using the statistical and graphics R software (http://cran.r-project.org/; version 3.1.3; 2015-03-9). A Shapiro-Wilk test of normality (Type I error criterion set to α=0.01) was performed on each of the 48 seed protein and oil phenotypic distributions. A Pearson correlation coefficient for protein and oil was also computed for each population.

Individual F_2_ plants (and the F_3_ seed progeny each produced) were the experimental units in this experiment. Because F_2_ plants cannot be naturally replicated to obtain an estimate of environmental variance, NIR assays were performed on the seed progenies harvested from the multiple homozygous female and male parent plants that had been grown in interspersed sections of the same nursery row containing F_2_ plants. Parental assay data were used to obtain an indirect estimate of the environmental variance using the following equation:

$${\text{σ}}_{\text{e}}^{2}=(1/2)({\text{σ}}_{\text{pFem}+}^{2}{\text{σ}}_{\text{pMal}}^{2})$$

where ${\text{σ}}_{\text{pFem}}^{2}$ and ${\text{σ}}_{\text{pMal}}^{2}\;$were the respective phenotypic variances in the seed protein for the seed produced by the high protein female parent, and by the high yield (but ordinary protein) male parent, respectively. The genetic variance component of the F_2:3_ progeny phenotypic variance was then estimated by subtraction, using this formula:

$${\text{σ}}_{\text{g}}^{2}={\text{σ}}_{\text{p}}^{2}-{\text{σ}}_{\text{e}}^{2}$$

where ${\text{σ}}_{\text{p}}^{2}$ was the F_2:3_ progeny phenotypic variance.

A broad sense heritability (H^2^) estimate was then obtained in the usual manner for each of the 48 populations (Bernardo 2010):

$${\text{ H}}^{2}=\frac{{\text{σ}}_{\text{g}}^{2}}{{\text{σ}}_{\text{p}}^{2}}\;\times 100\%$$

### QTL analysis

The R/qtl software package (http://www.rqtl.org/) was used in this study. A *.csv file containing phenotypic and genotypic data in a R/qtl csvr format was prepared for each of the 48 populations, and then error-checked prior to the QTL analysis (for details, see File S1). The maximum likelihood method of interval mapping, using the Expectation-Maximization (EM) algorithm, as implemented in R/qtl, was used for QTL detection (Xu and Vogl 2000; Sen *et al.* 2009). Estimates of chromosomal QTL map positions in each of the 48 populations were obtained not only for the SG trait of seed protein, but also for the non-SG trait of seed oil, primarily because of the well-known coinheritance of these two negatively correlated traits. With SG, stratified permutation testing was necessary (Manichaikul *et al.* 2007), and was applied to just the 44 genotyped F_2_ progenitors of the selected F_2:3_ progeny (*i.e.*, 22 low/22 high protein phenotypes) to obtain a (QTL peak) LOD score significance criterion for a *genome-wise* Type I error α of 0.05 +/− SE of 0.005. To attain this degree of precision (see p. 106 in Broman and Sen 2009), 1900-replicate permutation tests were conducted for each trait in each population. The protein (or oil) additive (*a*) and dominance (*d*) effects conditioned by each marker on each chromosome were first examined graphically, but subsequently, these two effects were numerically estimated for just the putative QTL exhibiting the largest peak LOD score on each chromosome. This estimation used the phenotype means for each of the A, H, and B genotypes of the SNP marker, or a pseudo-marker nearest to the putative QTL. The heritability of each presumptive single QTL on a chromosome is the fraction of the phenotypic variance (*i.e.*, *R*^2^) explained by that QTL, which was estimated with the following equation (p. 77 in Broman and Sen 2009):

$$R^{\text{2}}=\text{1}-{\text{10}}^{\left(-\text{2}/n\right)\text{LOD}}$$

where *n* is the number of phenotyped F_2:3_ progenies in each population, and LOD is the log_10_ likelihood ratio (LR) attained by that QTL at its peak map position in the Rqtl scanone output.

The QTL detected in this study were declared statistically significant only if the observed peak LOD score exceeded a population-specific, permutation-generated LOD score computed for a genome-wise Type I error of α = 0.05. The chromosomal locations of these QTL were compared with the locations of QTL detected using GWAS in the recent reports, and also the QTL detected in older publications listed in SoyBase. In the latter reports, the authors often used a lower significance threshold for QTL declaration (*i.e.*, LOD ≥ 3.0), which in most cases was also a comparison-wise threshold that was not adjusted for multiple testing.

### Data availability

Phenotype and genotype data for the 48 F_2_ populations and three combined sets of F_2_ populations (*.csv files) will be available on SoyBase (www.soybase.com), along with the R/qtl command code (*.txt files). Supplemental files include: File S1 contains additional *Materials and Methods* details; Figure S1 illustrates genetic diversity for seed protein/oil in the Soybean Germplasm Collection; Figure S2 shows the chromosomal map positions of the 1536 SNPs, and the 452 SNPs in the (example) SG mating 1; Figure S3 depicts the chromosomal map positions of SoyBase-listed QTL reported to date; Table S1 documents the original identification codes for the 1536 SNPs aligned with the shorter five-digit Snnnnn names we used to reduce computer memory usage, and to lessen printed table space in this report; Table S2 and Table S3 contain population-specific data for the respective phenotypic and genotypic data after R/qtl error-checking; Table S4 contains the parameter data derived from the population-specific QTL analyses, ordered by either mating number or by chromosome number; Table S5, Table S6, and Table S7 tabulate the QTL analysis information generated in the combined sets of parental matings of MG 000, 00, and 0 in which the high protein accessions were not uniquely different from each other in terms of SNP genotype.

## Results

**Figure 1:**
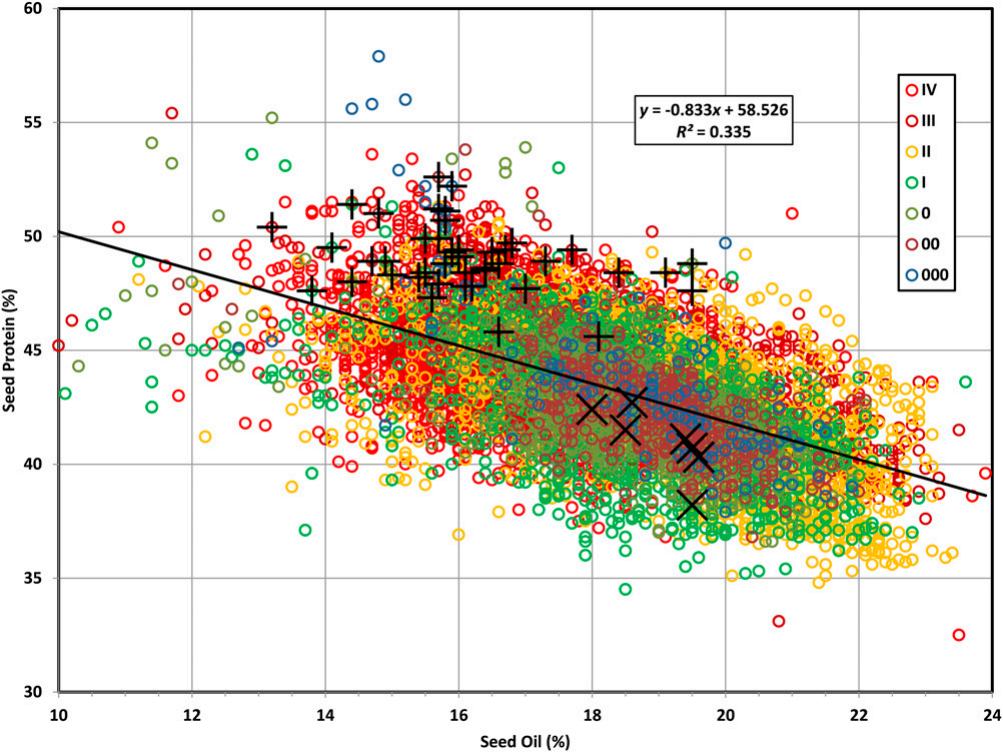
Seed protein values plotted against corresponding seed oil values. These are the GRIN values for 10,762 of the 17,711 *Glycine max* (L.) Merr. accessions in the USDA Soybean Germplasm Collection (as of December 31, 2015) in the seven maturity groups (MGs) of 000 (130), 00 (491), 0 (1179), I (1600), II (1831), III (1731), and IV (3800). Also shown are graph coordinates for 47 of the 48 high protein female parents (+), and seven agronomic male parents (×) used in this study (see Table 1).

A total of 48 high seed protein soybean accessions were used as female parents in this research (Table 1). Additional high protein accessions have since been added to the germplasm collection, though the 48 used here remain representative of the current group of such accessions (Figure 1). The male parent accessions (*i.e.*, high yielding cultivars of ordinary seed protein content) have a seed oil content that is characteristically higher than that of most of the female parents. Accessions with a maturity greater than MG IV (except for one very early maturing MG V) were not used in this study because the normal fall frost date in Lincoln, NE precludes completion of their normal seed maturation.

Our initial goal was to generate at least 250 F_2:3_ seed progenies in each mating, which was reached in most matings (Table S2), but not in some later MGs, though sufficient F_2_ plant numbers were raised per mating. Progeny numbers averaged 224 over the 48 matings, but ranged from 278 to 115. In later MG matings, many F_2_ plants produced too few F_3_ seed (due to pod shattering) to meet the minimum seed sample requirement of the NIR instrument.

### Phenotype data

The F_2:3_ seed protein distributions (Table S2), only three of the 48 seed protein distributions had *P*-values for the Shapiro-Wilkes normality test that were less than the prechosen criterion of *P* = 0.01 (*i.e.*, mating 31, *P* = 0.008; 38, *P* = 0.00005; 47, *P* = 0.003), primarily because of a rightward skew (perceptibly slight in matings 31 and 47, but notably more so in 38). Seven other distributions had *P*-values of less than *P* = 0.05, but these seven were still greater than *P* = 0.01 (*i.e.*, matings 3, 6, 9, 10, 12, 21, and 37).

In the one replicate F_2:3_ seed protein distributions, the minimum and maximum values among the 48 matings ranged from 371 to 402 g kg^–1^, and from 446 to 497 g kg^–1^ (Table S2). The F_2.3_ progeny seed protein means in those 48 matings ranged from 411 to 439 g kg^–1^.

### Heritability

The seed protein phenotypic variance in the 48 matings ranged from 13 to 53, with a mean of 27 (Table S2)—typical magnitudes when protein content is NIR-measured using F_3_ seed (*i.e.*, F_3_ embryos with F_2_ seed coats) produced by F_2_ plants derived from matings of high protein parents with ordinary protein parents. The F_2_ plant phenotypic variance, when divided by the summed parental plant phenotypic variances, led to moderately sized heritability estimates that averaged 66%, but ranged by mating from 30% to 87%. The seed oil phenotypic variance ranged from 12 to 58, with a mean of 26 (Table S2), and the heritability estimates (except for zero in mating 48) averaged 68%, and ranged from 18% to 93% in the other 47 matings. These estimates are based on just one (complete) replicate assay, one location, and one year, and thus do not have the accuracy of multi-environment-based heritability estimates (Visscher *et al.* 2008).

### Population SNP genotyping numbers

The SG percentage of the 44 genotyped population individuals was actually a function of the number of phenotyped individuals which, in any given mating, deviated from a 48-mating average of *n* = 224. The SG two-tail percentage averaged 20.5% (Table S2), though that percentage by mating varied from 15.8% (*i.e.*, mating 16) to 38.3% (*i.e.*, mating 45).

A majority of the SNPs (*ca*. 60%) in the 1536-SNP chip developed by Hyten *et al.* (2010) were not bimorphic in each of the 48 matings (Table S3). The 48-mating average for parental SNP bimorphism was 29.3% of the 1536, but, on an individual mating basis, ranged from 16.9% (mating 31) to 36.5% (mating 40). On a chromosome basis, the range was 24% (chromosomes 6 and 7) to 38% (chromosome 16). In a few matings, some chromosomes had fewer than 10 bimorphic SNPs, primarily because of the removal, during error-checking, of several problematic SNPs that, when paired with other nearby SNPs, generated recombination fraction values far above the expected 0.50 maximum.

Version 4.0 of the soybean genetic map spans 2296.4 cM (Hyten *et al.* 2010), but, if restricted to just the 1536 SNPs, the map is shorter (*i.e.*, 2156.2 cM). A 5-cM SNP spacing is considered to be sufficiently dense for optimizing QTL detection power in populations of size 200 (Strange *et al.* 2013), implying that 440–460 evenly spaced SNPs would thus be adequate for a 2150–2300 cM map. The mean number of SNP markers segregating per population in this SG study was, in fact, 450 (Table S3), but ranged from a maximum of 560 (mating 40) to a minimum of 259 (mating 31). The number of genotyped SNPs was low in two other cases (317 in mating 7; 305 in mating 8), but 396 or more SNPs did segregate in 40 of the 48 matings, with 348 SNPs or more segregating in five of the remaining eight matings. The 1536-SNP chip was designed to position SNPs as uniformly possible over the chromosomes (Figure S2A), but less than *ca*. one-third of those SNPs segregated in any given mating. An example is mating 1, in which only 452 SNPs were bimorphic (Figure S2B). Marker monomorphism did result in SNP-coverage gaps of 30 cM or more in some matings (in the mating 1 example, chromosomes 1, 6, 7, 12, and 20), but marker gaps are not *a priori* predictable when using a SNP chip for genotyping in a multi-mating SG strategy.

### QTL identified for seed protein and oil

**Figure 2:**
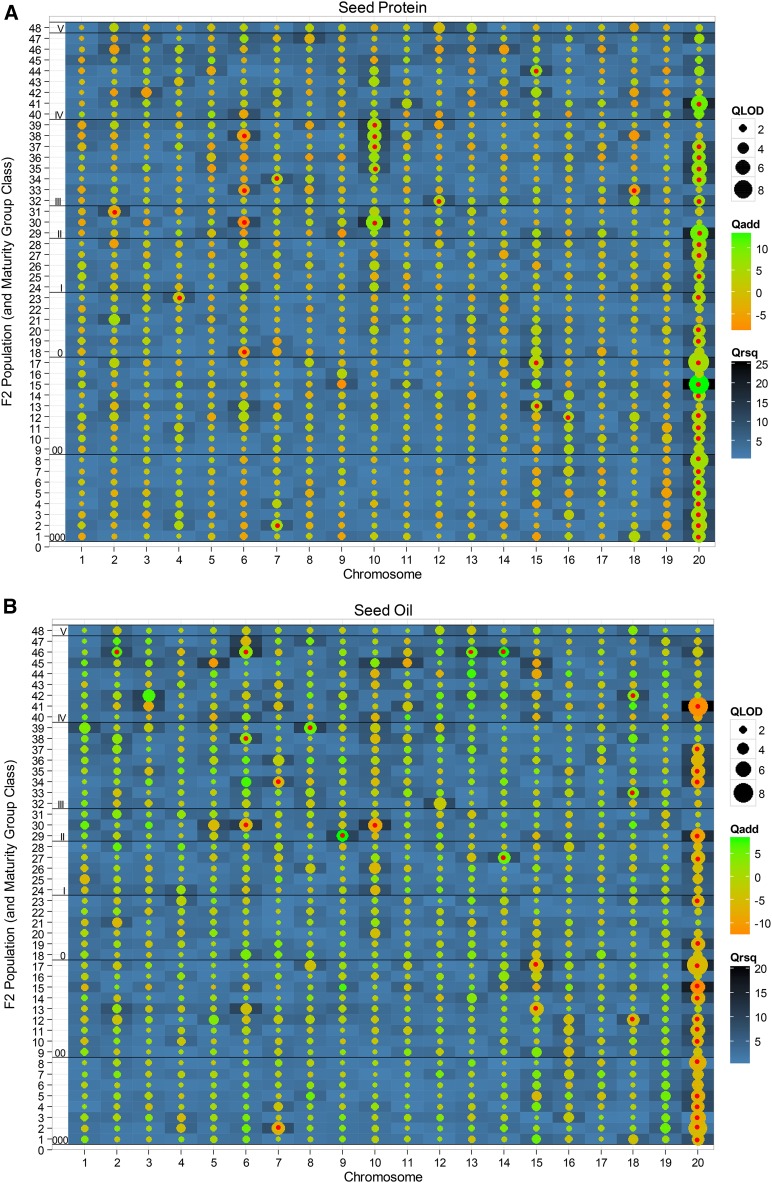
A heat map depicting parameter estimates for the SG-detected QTL for protein (A) and oil (B). The 48 MG-class matings are listed on the left axis, and 20 soybean chromosomes on the horizontal axis. The LOD score peak magnitudes are denoted by bubble size; those exceeding a genome-wise α = 0.05 significance threshold derived from trait- and population-specific SG-stratified permutation tests (*n* = 1900) have red dot centers. Additive effect magnitude is denoted by bubble color intensity; green denoting a positive and orange a negative directional effect of the female parent B allele. The magnitude of the *R*^2^ values is denoted by square tile color (light blue to deep black). See Table S4 for numerical values of QTL analysis parameters and permutation values.

The QTL analysis data obtained for soybean seed protein and seed oil in each of the 48 matings (Table S4) were translated into a heat map (Figure 2) to display the QTL peak LOD scores observed for seed protein (Figure 2A) or oil (Figure 2B) on any given soybean chromosome in each mating. The permutation-derived LOD score significance criterion (*i.e.*, genome-wise α of 0.05) for evaluating those observed QTL peak scores varied by population from 3.2 to 4.6 for protein, averaging *ca*. 4.0, and, for oil, varied from 3.2 to 5.6, also averaging *ca*. 4.0 (Table S4). Using the stratified permutation-based significance criterion, a QTL was detected on at least one chromosome for the SG trait of seed protein in 35 (73% of the 48) matings, and detected for the non-SG trait of seed oil in 25 (52% of the 48) matings (Figure 2, A and B, red-center bubbles). In two of the 48 matings (*i.e.*, 22 and 45), LOD score values on all 20 chromosomes were < 3.0, indicating the absence of any protein or oil QTL.

The LOD score heat map makes evident the near-ubiquitous segregation of the well-known chromosome 20 QTL for protein and/or oil in many SG matings. The protein QTL was significant in 27 (77%) of the above-noted 35 matings (*i.e.*, 56% of all 48) (Figure 2A), with the oil QTL being significant in 20 (80%) of the above-noted 25 matings (*i.e.*, 42% of all 48) (Figure 2B). Significant protein QTL were also detected on chromosome 10 (Figure 2A) in five matings (*i.e.*, 30 of MG II; 35, 37, 38, and 39 of MG III), but only in one mating (30), was a significant colocalized oil QTL detected (Figure 2B). The QTL region on chromosome 20 is known to be highly homologous with the long arm of chromosome 10 (Schmutz *et al.* 2010). Diers *et al.* (1992) reported that protein-oil QTL existed on chromosome 20 and 15. A protein and oil QTL was SG-detected on 15 in two MG 00 matings (13 and 17), but only for protein in MG IV mating 44 (Figure 2). Other less common SG-detected QTL were on chromosome 6 for protein (matings 18, 30, 33, and 38) and oil (30, 38, and 46), on 7 for both protein and oil (matings 2 and 34) and 18 (33), but just oil on 14 (27 and 46), and 18 (12, 33, and 42). Significant QTL were detected on chromosomes 2, 4, 12, 16, and 18 for protein, and on chromosomes 2, 8, 9 and 13 for oil, but only in single (separate) matings.

With respect to the significant seed protein QTL on chromosomes 20, 10, and 15, plus the protein QTL on chromosome 7 (matings 2 and 34), the QTL allele contributed by the high protein parent *enhanced* protein content, but coordinately *decreased* oil (Figure 2, A and B; +/− additive effects are denoted by a green/orange bubble color). Conversely, for the protein QTL repeatedly detected on chromosome 6 (matings 18, 30, 33, and 38), plus the protein QTL on chromosomes 2 and 18 (matings 31 and 18), the high protein parent allele *decreased* protein but *enhanced* oil.

For those significant protein and oil QTL that had coincident map positions, the protein and oil additive effects were directionally inversed (cf. Figure 2, A and B, and Table S4). Fewer oil QTL than protein QTL were detected, but this was expected, due to a SG focus only on protein, and a protein-oil correlation in the SG matings that, while strong, was clearly not unity, ranging from –0.66 to –0.88, averaging –0.78 (Table S2). Chung *et al.* (2003) noted the chromosome 20 segment had opposite effects on protein and oil contents, perhaps due to pleiotropy. A single-QTL pleiotropy hypothesis is easily falsifiable upon detection of a recombinant with a coupling- (instead of a repulsion-) phased phenotype, but no recombinant individuals with a *high protein*–*high oil* seed content were detected in this study.

## Discussion

Selective genotyping was a term first defined and used by Lander and Botstein (1989), though the method had been essentially described earlier by Lebowitz *et al.* (1987) as a “trait-based QTL analysis”, in which genotyping resources could be more efficiently allocated, with minimal loss of information, to just a fraction of progeny in a given mating. Indeed, Navabi *et al.* (2009) used simulation to document that, if 30 to 50 of 200 phenotyped progeny of a mating were genotyped in a bidirectional SG, Type I error would not exceed 0.02. With 20% genotyping, QTL detection power was still nearly 0.8 (*i.e.*, a Type II error of 0.20), though detection of QTL of moderate to large effect size would require a marker spacing of at least 5 cM. These results led Navabi *et al.* (2009) to conclude that SG would be a very effective tool for screening large numbers of potential donors for large-effect QTL alleles governing a particular trait of interest.

That strategy was evaluated here by genotyping *ca*. 20% of *ca*. 224 phenotypes in each of the 48 F_2_ populations created by using 48 high protein donor parents. We calculated, using the R program qtlDesign (Sen *et al.* 2007), that with a 5-cM SNP spacing, a Type I error (α) set to 0.05, and a Type II error (β) set to 0.2 to achieve a power (1 – β) of 0.8, QTL with an additive effect size of 5 g kg^–1^ (accounting for *ca*. 15% of the phenotypic variance) could be detected in such populations. In our 48-mating SG study, for which the significance threshold (genome-wise Type I error of 0.05) in each population was obtained by permutation (*n* – 1900), significant QTL with additive effects of ≥ 4 g kg^–1^, and *R*^2^ values of 0.07 or more, were detected for protein on 10 chromosomes (*i.e.*, 1, 4, 6, 7, 10, 12, 15, 16, 18, and 20; Figure 2A), and for oil on 11 chromosomes (*i.e.*, 2, 6, 7, 8, 9, 10, 13, 14, 15, 18, and 20; Figure 2B, and see Table S4 for QTL summary data), confirming that multiple donor parents can be successfully surveyed for QTL presence using a SG strategy.

Seed protein and oil QTL detected in biparental matings in older publications are summarized in SoyBase (www.soybase.com). The QTL ANOVA F-statistics in old reports are not convertible into LOD scores, but the LOD scores in more recent reports are convertible into an F-statistic (Broman and Sen 2009), so we graphed the ANOVA F-statistic *P*-value (*y*-axis) and map position (*x*-axis) of each SoyBase-reported QTL (Figure S3). The evidence for a SoyBase-reported QTL using these comparison-wise *P*-values ranged from “merely suggestive” (*i.e.*, *P* < 0.01 = 10^−2^)—a significance criterion leading to a naïve supposition that a SoyBase-listed QTL exists on every soybean chromosome (except 16 for protein), to “highly likely” (*i.e.*, *P* < 0.0001 = 10^−4^)—a stringent significance criterion that offsets an intrinsic multiple marker comparison-wise test problem in the older reports. Using the latter criterion, we filtered the SoyBase-reported QTL to just the “most likely” protein QTL on the eight chromosomes of 1, 4, 6, 7, 11, 13, 15, and 20, and the oil QTL on the 10 chromosomes of 2, 5, 6, 8, 11, 14, 15, 16, 19, and 20 (Figure S3), wherein the underscores denote chromosomes in common with those having SG-significant protein or oil QTL (Figure 2). Comparatively, the SG study did reidentify some prior reported QTL; however, none of the 48 high protein SG donor parents were used in any of the 35 to 38 matings listed in SoyBase QTL reports, so this multi-mating SG strategy effectively doubled the number of biparental mapping populations used to date for detection of protein and oil QTL.

Korte and Farlow (2013) noted that GWAS surmounts two key limitations of biparental mapping: a QTL allele in a large-accession GWAS is not restricted to a 0.5 or zero frequency, as might be the case in any given biparental mating, and the QTL mapping resolution is greatly limited by the low number of potential recombination events in a F_2_ or RIL population, even if the latter were to be increased to *n* > 1000 individuals to boost the number of recombinant events. Though GWAS does require marker-dense genotyping (*i.e.*, thousands of SNPs) to achieve its signal resolution potential, those SNP numbers are nowadays more easily obtainable in soybean, given the availability of a 50K SNP chip (Song *et al.* 2013), or using genotyping-by-sequencing to generate, *de novo*, several thousands of SNPs (Sonah *et al.* 2014).

**Figure 3:**
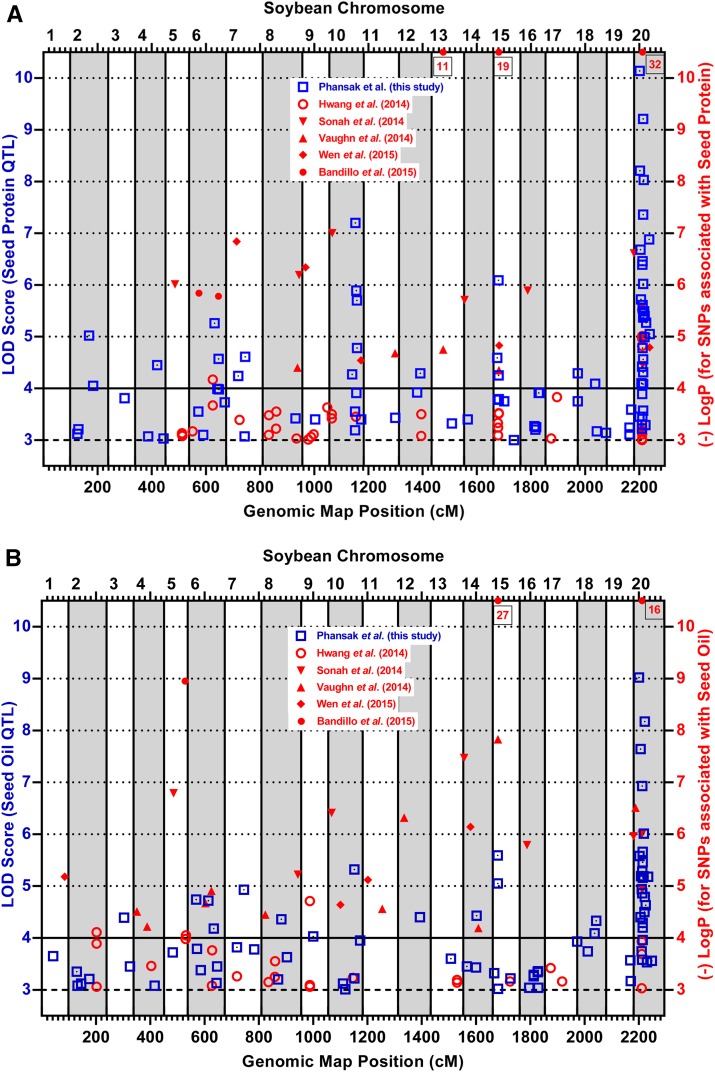
A graph of LOD score magnitudes of SG-detected QTL in 48 F_2_ populations for seed protein (A) and oil (B). The bottom axis is scaled in terms of the Version 4.0 cumulative genetic map positions in the 20-chromosome soybean genome. The blue-box symbols with centered blue dots denote SG QTL exceeding a genome-wise α = 0.05 significance threshold derived from trait- and population-specific SG-stratified permutation tests (*n* = 1900). Those thresholds varied from 3.6 to 4.6 for protein, and from 3.2 to 4.8 for oil, but averaged *ca*. 4.0 (horizontal black line). For comparative purposes, QTL detected in five recent GWAS publications are depicted relative to a –logP scaled right axis, though some Bandillo *et al.* (2015) values (box-enclosed at graph top) exceeded the scale limit.

The question then is whether a SG strategy is a worthy alternative to just using GWAS. Notably, biparental mapping and GWAS are still considered complementary approaches (Myles *et al.* 2009; Würschum 2012; Sonah *et al.* 2014). In fact, we considered our multi-mating SG strategy, wherein *ca*. 450 SNPs were used to genotype just the highest 22 (10%) and lowest 22 (10%) protein phenotypes in *ca*. 224 progeny derived from 48 high protein × low protein parental matings of MG 000 to IV to be contextually analogous to a phenotypic contrast type of GWAS [like the one recently conducted by Song *et al.* (2015) on soybean 100-seed weight]. The GWAS of Hwang *et al.* (2014) involved 31,954 SNP genotypes of 298 accessions of MG II, III, and IV, of which 151 had a high GRIN-based protein values, and 147 had a GRIN-based low protein values [though the contrasting GRIN values were only modestly (*r* = 0.6) correlated their field-based trial estimated values]. They detected significant QTL (using –logP ≥ 3) on *ca*. half of the chromosomes (Figure 3, A and B), and some of those QTL had chromosomal map positions coincident with some of our SG-detected significant QTL. Their two groups did include four SG high-protein female parents (32, 34, and 39 of MG III, plus 40 of MG IV), and two SG low-protein male parents (MGII Dwight and MG III Pana) (Table 1). Still, the comparative QTL results demonstrated that a SG survey strategy with *ca*. 48 accessions identified significant QTL with about the same degree of success achievable in a GWAS with *ca*. 300 accessions.

Recently, GWAS was used to detect seed protein and oil QTL (Sonah *et al.* 2014; Vaughn *et al.* 2014; Bandillo *et al.* 2015; Wen *et al.* 2015). The signal strength and map position of the significant QTL detected in our 48-mating SG can be compared to these GWAS-detected QTL (Figure 3). The –logP significance criterion / MG accession numbers / SNP numbers varied (*i.e.*, Sonah: 4/139 MG 0/17.2K SNPs; Vaughn: 4/619 MG I-II and 977 MG III–IV/∼32K SNPs; Bandillo: 5.7/12K MG 000 – X/36.5K SNPs; Wen: 5/1.4K MG I–III/3.75K SNPs), but in all cases, a minor allele frequency (MAF) cut-off of 0.05 was used. Accession number maximization is often sought in GWAS, because doing so increases historical recombinant event numbers, thus enhancing statistical power, and QTL signal resolution. However, if only a few accessions possess an allele of notable effect at a given QTL, nondetection of that QTL will occur in GWAS if those few accessions comprise less than a 0.05 fraction of all of the evaluated accessions. In fact, the routine use of MAF ≥ 0.05 in GWAS will, *a priori*, remove SNP locus alleles that are in complete linkage disequilibrium with a rare QTL allele that has an *in situ* frequency of < 0.05. Bandillo *et al.* (2015) documented this by showing that the high protein–low oil allele (of large effect) at the well-known chromosome 20 QTL was present in just over 1% of the 12K accessions they examined. But, when they parsed the 12K accessions into smaller groups, based on seven countries of origin, or on eight MG classes, the high protein allele on chromosome 20 had an MAF < 0.05 in all but the Korean accession subset (and in all but the MG V to X subsets). Sonah *et al.* (2014) and Wen *et al.* (2015) did detect the chromosome 20 QTL allele in their respective sets of MG 0 and MG I–III accessions, but Vaughn *et al.* (2014) did not in two large sets of MG I–II or MG III–IV accessions (Figure 3). Myles *et al.* (2009) commented on the ineffectiveness of GWAS relative to the detection of rare alleles, and noted that controlled crosses and family-based mapping would be needed to artificially inflate the infrequency of rare functional alleles to improve the power needed for their detection, and to thus better understand the role that rare alleles play with regard to heritability of a given trait of interest.

Despite its rarity, the chromosome 20 QTL was obviously detected in many of the 48 MG 000 to IV donor parent accessions surveyed in this SG study (Figure 2). Accessions chosen for a SG-based QTL survey are actually quite likely to possess rare QTL alleles of a large-to-moderate effect in heritable traits, given the use of an “extreme” phenotype criterion to select SG donor accessions. If at least one chosen donor parent accession possesses a rare allele, its frequency will obviously be 0.5 in the progeny of the corresponding SG biparental high × low mating, thus empowering its detection as noted by Myles *et al.* (2009).

A multi-mating F_2_ population SG strategy can provide multiple estimates of the additive (and dominance) effects for the significant QTL detected in more than one mating. However, our population sizes were *ca*. 224 in size, and thus the effect estimates are likely overly optimistic, and selectively biased (Beavis 1998; Xu 2003; Broman and Sen 2009). For greater precision and accuracy in effect estimation, a fivefold (or greater) population size is needed, which, along with a more marker-dense SNP chip for genotyping (Strange *et al.* 2013), potentially mitigates SNP-to-SNP marker linkage map gaps. Despite that problem, our foremost objective in this SG study was evaluating an economical means for *per se* detection of significant protein and oil QTL in a large potential donor accession set. Using GWAS instead of SG offers no panacea for better estimation, given that effect estimates are always specific for the reference population used in either approach, as noted by Würschum (2012). Breeders must obviously conduct follow-up research to precisely estimate the QTL allele effect size in the genetic backgrounds of their particular high-yielding cultivar sets, and to determine the worthiness of launching any marker-assisted high protein allele introgression program.

Song *et al.* (2015) found, after conducting a pairwise genetic similarity analysis using the 50K SNP chip, that 9% of the 18,480 accessions in the soybean germplasm collection had SNP genotypes that were not unique. They also reported that, using a 99.9% similarity criterion, 23% could be considered to be not unique. That discovery prompted us to review the 50K SNP genotypes of our 48 accessions. Unfortunately, eight of our MG 000 high protein accession parents (matings 1 to 8 in Table 1), and five of our MG 00 high protein parental accessions (matings 9–12 and 14) were not unique. Four MG 0 parental accessions (matings 18–20 and 22) also were not unique, but these four did differ from the former 13 accessions. Thus, only 33 of our 48 accessions were truly unique. Soybean breeders have used these MG 000, 00, and 0 accessions as a source of high protein alleles (Table 1), generally presuming that their differing GRIN passport data implied source diversity, but the germplasm SNP genotyping data reveals this presumption was mistaken.

The nonuniqueness of 13 MG 000 and 00 accessions, and the four MG 0 accessions, was a disappointing discovery, but it did offer a serendipitous opportunity to conduct a QTL analysis on three large biparental F_2_ populations obtainable by pooling the *ca*. *n* = 224 F_2_ populations of the three parental mating sets (*i.e.*, eight in MG 000, five in MG 00, and four in MG 0) based on the mating of those three sets of female parents to differing MG 000, 00, and 0 male parents. The pooled F_2_ phenotype numbers were respectively 2052, 1249, and 986. Soybean populations of this size have not, to our knowledge, been reported for biparental QTL mapping studies, and thus could be used to obtain more precise estimates of QTL peak map positions and, because of the large population sizes, the inflationary impact of selection bias on allele effect estimates would be mitigated (Broman and Sen 2009).

**Figure 4:**
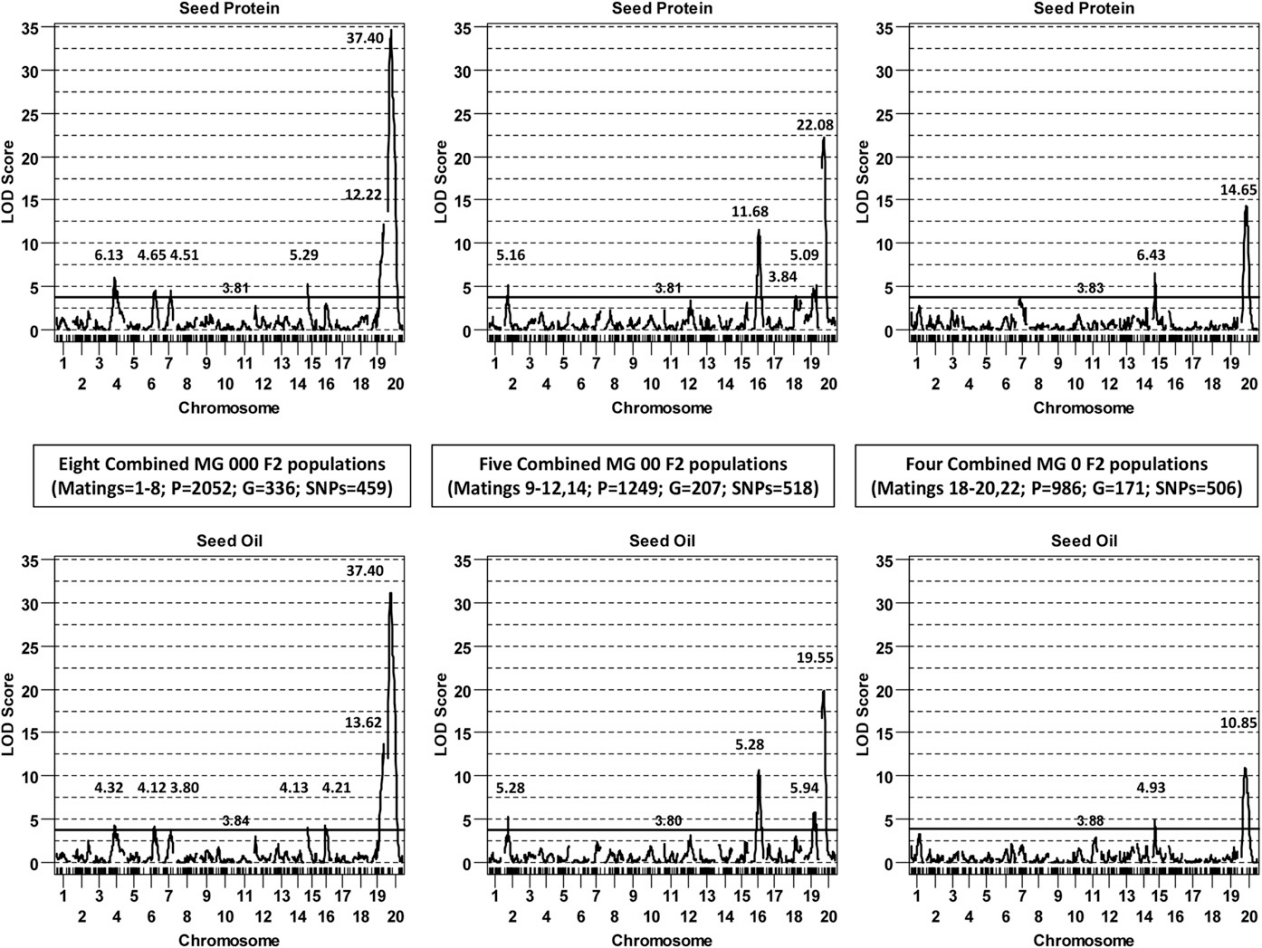
Chromosomal LOD score scans for protein (top panels) and oil (bottom panels). Selectively genotyped F_2_ populations derived from parental matings in which the high protein accessions were not unique in terms of SNP genotype were pooled into three MG sets of 000 (left panels), 00 (middle panels), and 0 (right panels). The SG percentages were a respective 16.4, 16.8, and 17.3%, relative to the numbers of phenotypes (P), genotypes (G), and bimorphic SNPs shown for each MG set. Genome-wise α = 0.05 significance thresholds, derived from trait- and population-specific SG-stratified permutation tests (*n* = 1900), were nearly-identical (*i.e.*, the 3.80 to 3.88 threshold horizontal lines shown in each panel).

The well-known major QTL located at the proximal end of chromosome 20 was detected in each pooled MG set (Figure 4,), and also in the *ca*. *n* = 224 populations, except mating 9 in MG 00 and mating 18 and 22 in MG 0 (Figure 2; for details see Table S5, Table S6, and Table S7). In contrast, QTL were detected on chromosome 19 in the MG 000 and 00 sets (Figure 4), but were not detected in any small population comprising those two sets (Figure 2). Similarly, the chromosome 4, 6, 7, and 15 QTL detected in MG 000 were not detected in small populations (except on chromosome 7 in mating 2). Finally, the QTL on chromosomes 2, 16, and 18 that were detected in MG 00 were not detected in small populations (except for mating 12 on 16 and 18 – oil only). These QTL likely had modest allelic effects that did not exceed the QTL detection limit in the small populations (*i.e.*, equivalent to false negatives), but did exceed it in the 5- to 10-fold larger populations.

**Figure 5:**
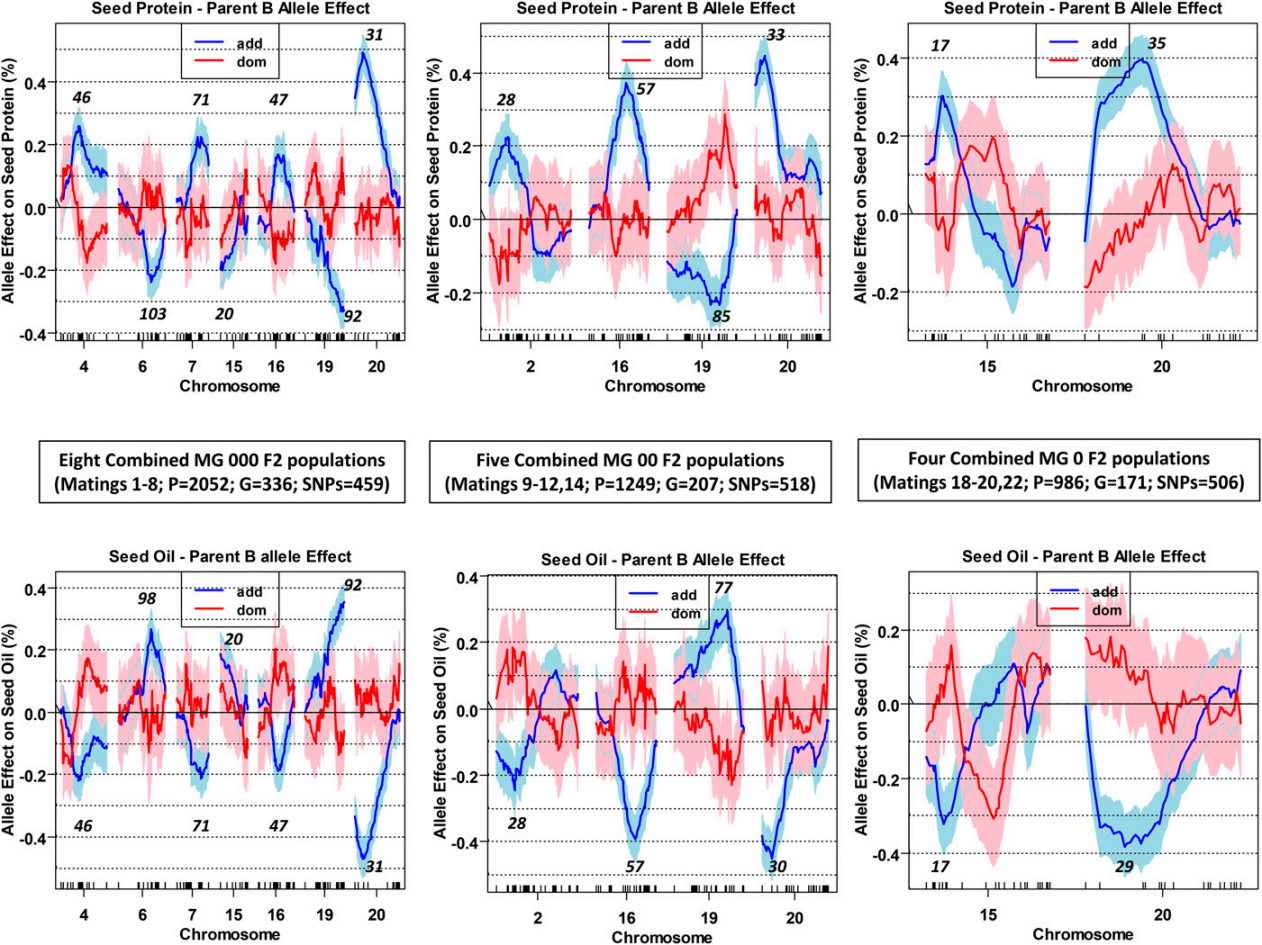
Chromosomal scans of the estimated additive and dominance effects for protein (top panels) and oil (bottom panels). The scans were limited to just the chromosomes exhibiting LOD score peaks shown in Figure 4. Effect magnitude and direction (+/−) reflect the substitution of a female parent B allele for a male parent A allele at any given bimorphic SNP position, with shading denoting the SE of the effect mean at each SNP. The chromosomal cM positions of the positive and negative additive effect maxima are italicized.

By using chromosome-specific R/qtl additive and dominance effect scans (Figure 5), one can graphically view the impact of substituting a female parent B allele for the male parent A allele at each successive SNP on a chromosome. Coincident map positions were evident for most of the same-chromosome protein and oil QTL peaks, with such numbers being more concordant with a 1-locus pleiotropy than a 2-locus linkage model (Chung *et al.* 2003). The allele contributed by the high protein parents for the chromosomes 2, 4, 7, 16, and 20 QTL enhanced protein but decreased oil, whereas the allele contributed by the same parents for the chromosome 6 and 19 QTL decreased protein but enhanced seed oil. One peculiarity in these scans was the differential additive effect scan patterns for the chromosome 15 QTL detected in MG 000 *vs.* MG 0 (Figure 5). The eight high protein female parents in the MG 000 set contributed an allele that decreased seed protein, whereas the four MG 0 high protein female parents contributed an allele that enhanced seed protein. The *trans*-phased phenotypic effect at these two linked QTL (*i.e.*, located at 20 cM in MG 000, but at 17 cM in MG 0) is notable, even if different male parents were used in these MG sets. We are not aware of any soybean linkage mapping study or GWAS documenting a similar *trans*-phased QTL pair.

The tracking of QTL dominance and additive affects in the three MG sets revealed that, at each chromosomal QTL (Figure 5, Table S5, Table S6, and Table S7), when the additive effect was positive, the dominance effect was typically (though not always) negative, and *vice versa*. However the SE boundary for the additive effect was narrower than that bounding the dominance effect—an indication that the latter was less precisely estimated, likely due to heterozygote infrequency in SG phenotypic extremes. Ordinarily, additive, plus additive × additive epistasis, accounts for most of the total trait genetic variance in soybean (Burton 1987). Only inbred cultivars are used in commercial production, and the creation of F_1_ hybrids is not likely anytime soon. Dominance effects would more likely be of breeder interest if made available for yield rather than seed protein and oil.

The discovery, or confirmatory rediscovery, of protein and oil QTL and map positions in this SG-based survey of high protein donor accessions will likely to be of relevance to soybean breeders. The SG survey strategy did identify “major” protein and oil QTL in the 48 donor accessions examined here, suggesting that it could be used detect to major QTL alleles (and potentially rare ones) in traits other than seed composition, assuming that such traits can be reliably quantified using individual F_2_ plants. A major drawback to the SG strategy is the need to apply it to phenotyping populations much larger than the *n* = 224 size used in this study, if the goal is to detect QTL of more modest additive effect.

## 

## Supplementary Material

Supplemental Material
