# Supplementary material for: Multi-Population Selective Genotyping to Identify Soybean [Glycine max (L.) Merr.] Seed Protein and Oil QTLs
Source: G3 (Bethesda). 2016 Apr 1;6(6):1635–48. doi: 10.1534/g3.116.027656 (PMC4889660; doi:10.1534/g3.116.027656)
Supplement: Supplemental Material [file supp_6_6_1635__index.html]

Supplemental Material 

# Multi-Population Selective Genotyping to Identify Soybean [*Glycine max* (L.) Merr.] Seed Protein and Oil QTLs

Supplemental Material for Phansak *et al.*, 2016

Supplemental Material

**Files in this Data Supplement:**

- File S1 - Materials and methods. (.pdf, 462 KB)
- Figure S1 - Soybean seed protein and oil values plotted against the corresponding seed yield values for maturity group (MG) 0 to IV accessions in the [*Glycine. max*(L.) Merr.] germplasm collection. (.pdf, 392 KB)
- Figure S2 - SNP marker map position (cross-hairs) in the 20 soybean chromosomes for (A) all of the 1536 SNPs in the chip developed by Hyten *et al*.(2010) and (B) just the 452 SNPs segregating in (the example) mating 1 of the 48 F2populations examined in this study. (.pdf, 39 KB)
- Figure S3 - A graph of ANOVA F-statistic probabilities for (A) seed protein QTLs and (B) seed oil QTLs listed in SoyBase (Grant *et al*.2010). (.pdf, 23 KB)
- Table S1 - Translation table relating the 1536 SNP BARC ID# numbers (as listed in Soybase) to a synonymous but shorter S-prefixed. (.xlsx, 141 KB)
- Table S2 - F1 , F2, F1:2 and F2:3 plants, seeds, and progeny numbers in each of the 48 matings, ordered by MG then by mating code and ID number. (.pdf, 105 KB)
- Table S3 - The number of SNP markers detected as segregating in each soybean chromosome in each of the 48 F2 populations *versus* the 1536 potentially detectable SNPs on the USLP 1.0 chip (Hyten *et al*. 2010). (.pdf, 140 KB)
- Table S4 - Population-specific QTL analysis parameters for soybean seed protein (left) and oil (right) arranged by mating number (1-48). (.xlsx, 509 KB)
- Table S5 - QTL analysis parameters for seed protein and oil in F2 populations of non-unique MG 000 SG matings of 1 to 8 (see Table 1). (.xlsx, 22 KB)
- Table S6 - QTL analysis parameters for seed protein and oil in F2 populations of non-unique MG 00 SG matings of 9 to 12 & 14 (Table 1). (.xlsx, 17 KB)
- Table S7 - QTL analysis parameters for seed protein and oil in F*2* populations of non-unique MG 0 SG matings of 18 to 20 & 22 (Table 1). (.xlsx, 15 KB)
