# Supplementary material for: Multi-Population Selective Genotyping to Identify Soybean [Glycine max (L.) Merr.] Seed Protein and Oil QTLs
Source: G3 (Bethesda). 2016 Apr 1;6(6):1635–48. doi: 10.1534/g3.116.027656 (PMC4889660; doi:10.1534/g3.116.027656)
Supplement: Supplemental Material [file supp_g3.116.027656_FileS1.pdf]

Multi-Population Selective Genotyping to Identify Soybean

(*Glycine max* (L.) Merr.) Seed Protein and Oil QTLs

Piyaporn Phansak[*,1], Watcharin Soonsuwon[*,2], David L. Hyten[*], Qijian Song[§],

Perry B. Cregan[§], George L. Graef[*], and James E. Specht[*]

*** Department of Agronomy & Horticulture, University of Nebraska, Lincoln, Nebraska, 68583-0915, USA. § USDA-ARS, Soybean Genomics and Improvement Laboratory, Beltsville, MD, 20705-2325, USA. 1 Current Address: Division of Biology, Faculty of Science, Nakhon Phanom University, Muang, Nakhon Phanom, 48000 Thailand. 2 Current Address: Department of Plant Science, Faculty of Natural Resources, Prince of Songkla University, Hat Yai, Songkhla, 90112 Thailand.**

Corresponding author e-mail: jspecht1@unl.edu

FILE S1

MATERIALS AND METHODS

Leaf Collection and DNA Extraction Procedures

Parents and F_1 Plants: One young trifoliate leaf was collected from each parental plant and from each F_1 plant (to be authenticated as to its hybridity) and placed into a deep 96-well collection plate that was stored at -20°C until subsequent DNA extraction. The DNA was extracted from the leaf tissue samples using a DNA extraction protocol described by (Saghai-Maroof *et al*. 1984), except that the volumes were halved. The DNA concentration of each sample was quantified using a spectrophotometer set to a wavelength of 260 and 280 nm. The stock DNA samples were diluted to 20 ng μL^{-1} for use in subsequent marker analyses.

F_2 Plants: A single newly developed trifoliolate leaf with leaflets about 2 cm in length was excised from the main stem apex of each tagged F_2 plant in each of the 48 population-specific nursery rows. The three excised leaflets were gently rolled between the forefingers into a tight bundle that was inserted into a labeled well of a deep-well tissue collection plate. Plates were kept on ice during leaf tissue sampling until all wells were filled with tissue, and then immediately placed into a -20°C freezer. After the completion of phenotyping and the subsequent identification of $F_{2:3}$ progenies in the lowest and highest decile fractions of the seed protein distribution in the fall of 2008, the leaf tissue samples of their F_2 plant progenitors were later retrieved from the collection plates and placed into 96-well extraction plates. Because of the cost of genotyping and the use of a 96-well plate format for genotyping, leaf samples of just the 22 F_2 plant progenitors of the seed progenies residing in the extreme decile seed protein phenotypic tails in each population were selected for genotyping. A 96-well extraction plate thus contained 47 leaf tissue samples of one given population (i.e., its 22 highest and 22 lowest protein F_2 plants, a confirmed F_1 plant, and the high and low protein parent plants), plus 47 leaf samples of the same type from another F_2 population. The two remaining wells were used for a repeat (insurance) sample of the high protein parent of each of the two populations. Plates were shipped on dry ice to a USDA laboratory in Beltsville, MD, for DNA extraction.

Genotypic Error Checking

The R package known as R/qtl (Broman *et al.* 2003; http://www.rqtl.org/) was used to error-check the phenotypic and genotypic data in each of the 48 populations, before that same software was used to conduct the QTL analyses. The phenotypic and genotypic data for each given population were organized into an Excel file in a comma-delimited “csvr” format (see Fig. 2.4, Broman and Sen, 2009). The 17-digit BARC number identifier of each of the 1536 SNPs was translated into a more compact S-prefixed 5-digit SNP ID# (Table S1), which minimized tabulation space requirements in the R/qtl output (and in the tables here).

The chromosome number and map position of each segregating SNP marker present in each *.csvr file were those published in the Version 4.0 soybean genetic map (Hyten *et al.* 2010).

A Chi-square goodness-of-fit test was conducted to identify allelic segregation distortion (R/qtl command: *geno.table*). Using a population-specific genome-wise alpha (generated by simply dividing a test-wise Type I alpha of 0.05 by the number of markers in each population), this test identified an average of 4-5 SNPs (range of 0 to 12) that were removed (*drop.markers*) from each population except mating 2 (Table 1), for which 57 such SNPs had to be removed.

A check for duplicate individuals (*comparegeno*) in each population identified a pair of F_2 individuals with 100% identical SNP genotypes in each of two populations (matings 11, 14), and a pair with 95% identity in another population (mating 10). These were likely the result of a harvest, threshing, or labeling error, so only the pair member with the greatest number of SNP marker genotypes was retained (*subset.cross*).

The crossover (XO) count for each genotyped F_2 plant was examined (*countXO*) to check for XO outlier counts that were abnormally high (n>175) or abnormally low (n<16) on a population-specific basis. On average, more than half of the 48 populations had no such outliers, and only 1 or 2 outliers were detected in the remaining populations (though six probable self progeny of the female parent were detected in mating 17). The few outliers were not retained (*subset.cross*).

Closely linked duplicate, triplicate, or quadruplicate SNP loci that did not recombine were identified as haplotype sets (*findDupMakers*; *exact.only*), and these di-, tri- , and quad sets of loci ranged from a respective 0 to 63, 0 to 18, and 0 to 4 in the 48 populations. This was not a surprising finding, given the limited opportunity for recombination events in these 44-genotype SG populations. In a single population QTL analysis, one ordinarily drops all but one member of a haplotype set. However, in this QTL analysis involving 48 populations, a slight difference (1×10^{-6} cM) in the map positions of the SNPs in each haplotype set was generated (*jittermap*) to retain these SNP markers for the purpose of inter-population comparisons of the markers near any QTL identified at a (near-) identical map position in at least two (or more) of the 48 populations

Allele codes for a given SNP marker can potentially be erroneously recorded in an inverted phase (i.e., A<>B). This condition may arise during the translation of base-pair SNP genotypic code into A-H-B marker genotypic code, usually when genotypic base-pair code is missing (or is heterozygous) for either (or both) inbred parent(s) for a given SNP marker. A range of 0 to 7 SNPs with such inverted allele code errors were detected (*checkAlleles*) in the 48 populations, though one population (mating 20) had 22 such potential errors. If the genotype counts (*geno.crostab*) for a marker pair involving the SNP with the potentially inverted alleles and either its left or right flanking marker provided inferentially sound evidence for doing so, then the erroneously phased allele code for that SNP was purposely re-phased (*switchAlleles*), but if not, then that SNP was dropped (*drop.markers*). The graphing of marker pair recombination fraction (rf) LOD scores against the corresponding rf values (*plot.rf*) also was used (1) to flag those marker pairs with a rf >0.85 (i.e., a criterion chosen based on the potential for an rf deviation of that magnitude from the theoretically expected rf=0.50 in a 44-genotype sample), and (2) to flag any other marker pairs with rf values of just less than 0.85 that had a rf LOD score >4.0. The majority of the marker pairs so flagged typically possessed the same common problematic marker member, and when that problematic marker was removed, the number of flagged pairs substantively decreased or went to zero.

Lincoln and Lander (1992) described an after-the-fact procedure for detecting apparent SNP genotyping errors, and because a variant of that *a posteriori* method is implemented in R/qtl (p. 381, Broman and Sen, 2009), it was used to evaluate the SNP genotype data in each of the 48 populations (*calc.errorlod*). The output list of potential genotyping errors (*toperrorlod; LOD>5*) contained, on average, about eight SNP markers per population (range of 0 to 24) that were present in multiple F_2 individuals (typically three to 20 or more), and these SNP markers were dropped (*drop.markers*). The remaining SNPs in the

outputted error list were not dropped; instead, in the one or two individuals identified with presumed SNP genotype errors, the SNP marker genotypes for those individuals were converted to missing (-) values.

The construction of an intrinsic linkage map for a typical SG population is problematic, not only because chromosomal marker order estimation is much less precise, but also because it is likely to be less accurate, due to the fact that genotype errors and missing SNP genotypes have a greater influence on marker order when the number of genotyped individuals is small (Martinez 1996). In this study, only 44 genotypes (or somewhat fewer, after error-checking) were available for marker order estimation in each population. For a QTL analysis, it is well known that marker order must be as correct as possible of avoid errors in the estimation of QTL map positions (p. 53, Broman and Sen 2009). Chromosomal marker positions can be reliably ascertained using a published genome sequence that has undergone multiple rounds of sequence error correction. Though very tightly linked SNP markers in the Hyten *et al*. (2010) Version 4.0 genetic map may still not be correctly ordered, the soybean breeding and genetics research community considers the linked markers in that map to be otherwise ordered to a sufficient degree of accuracy for mapping purposes. For that reason, we elected to use the Version 4.0 map of 1536 SNP loci as the chromosomal SNP marker order for each of our 48 populations. Population-specific recombination fractions were estimated (*est.rf*), as were the inter-marker map distances (*est.map,* e*rror.prob=0.001, map.function=kosambi*). The choice of 0.001 as an error probability parameter reflects an author conjecture of about one genotype error occurring per 1000 genotypes, thus implying that about 22 such errors would be expected per population amongst the approximate 22,000 genotypes generated when just 44 F_2 individuals per population are genotyped with *ca.* 500 SNPs.

The phenotype numbers and statistics after error-checking are presented in Table S2. A few F_2 progenitors had to be removed in some matings based on phenotypic errors, and, unfortunately, some of those that were dropped were members of the 44 selectively genotyped F_2 progenitors – six in three matings (9, 14, 46) and four in two matings (4, 23). In any event, the removal of a few F_2 plant phenotypes (genotyped or not) only slightly reduced the average SG percentage from 20.5 to 20%.

The genotype numbers and statistics after error-checking are presented in Table S3. The non-missing genotype percentage column in this table was calculated as the total number of SNP genotypes (AA, AB, BB) *minus* any missing ones (--), divided by total number of possible SNP genotypes (i.e., final SNP loci number multiplied by the final F_2 phenotype number). This parameter provided insight as to the degree to which the selectively genotyped individuals in each mating had missing genotypes at individual segregating SNP loci. The average over the 48 matings was 19%, which when compared to the 20% SG, indicates that SG individuals were not missing many SNP genotypes.

Relative to the collective AA: AB: BB genotype ratio (i.e., summed over all SNP loci segregating in a given mating), Chi-square tests (data not shown) revealed a good fit ($P>0.01$) of the observed ratio with the expected 1:2:1 F_2 genotype ratio in most matings (Table S3), though only marginally so in matings 6 ($P=0.038$), 41 ($P=0.029$), and 45 ($P=0.045$). However, a significant lack of fit ($P<0.001$) was evident in five matings, resulting from either an excess of AB heterozygotes (matings 2, 4, 8), or an excess of the male parent AA genotypes (matings 31, 32). Genes giving rise to gametophytic or zygotic differences in sterility, mortality, or vigor are typically the underlying causes of marker segregation distortion (SD). However, in these five populations, the SD was mostly genome-wide, as opposed to being limited to a specific localized chromosomal segment of SD that would arise if the SNPs therein were linked to a SD-causing genetic factor. Although SD can affect QTL detection power if the SD occurs in the closely linked flanking SNPs, Zhang *et al*. (2010) noted that the power can actually increase or decrease and, as long as the SD is not asymmetric to an extreme degree. They also noted that SD does not increase the number of false-positive QTLs, nor does it significantly impact the estimation of QTL position and effect.

For QTL detection with R/qtl, conditional genotypic probabilities were computed on a 2-cM grid basis (first with *calc.genoprob, step=2, errorprob = 0.001*, and thence with *sim.geno*, *step=2*, *n.draws=128*, *errorprob=0.001*), prior to conducting the interval mapping analysis (*scanone, method="em", addcov=ac, n.perm=1900, perm.strata=strat*). The additive covariate parameter was not used in the analysis of the 48 individual populations, but was used in the analysis of three F_2 progeny sets, wherein each set was comprised of F_2 populations derived from SNP-identical parental matings in MG 000, 00, and 0. The parameter *ac* was a mating number assigned to each of the F_2 populations present in the given set.

Permutation (1900 replicates) was performed on just the selectively genotyped 44 F_2 plant phenotypes and their genotypes (*strat*) in each mating to obtain population-specific LOD score criterion based on a genome-wise alpha=0.05. Permutation was similarly conducted in each of the three combined F_2 sets. The R/qtl output from *scanone* was used to compute a 95% Bayes credible interval for each detected chromosomal QTL in each F_2 population or each combined set (*bayesint, prob=0.95*). Numerical data for the protein and oil QTL peak parameters and permutation-based LOD threshold values that were generated with R/qtl are presented in Table S4, ordered by mating code and by chromosome number. Composite interval analysis (CIM) was not conducted with SG data generated in this study, because some experts express caution about using CIM when the missing genotype data is substantial (i.e., ca. 180 individuals were missing genotypes the ca. n=224 population sizes of the 48 matings. For one such expert view, see p. 206 of Broman and Sen (2009), and because SG requires a stratified permutation test (i.e., shuffling of phenotypes within just genotyped individual class separately from the non-genotyped class) for proper computation of a significance threshold LOD score (Manichaikul *et al*. 2007). The same argument applies for not conducting multiple qtl mapping with the SG data sets, given that multiple imputation can potentially result in spurious results when 80% of the genotypes are missing, i.e., p. 312 of Broman and Sen (2009). Attempts to apply the Blanc *et al.* (2009) connected population approach to our SG data sets were not successful, because of the substantial missing data.

LITERATURE CITED

Blanc, G., A. Charcosset, B. Mangin, A. Gallais, and L. Moreau, 2006 Connected populations for detecting quantitative trait loci and testing for espistasis: and application in maize. Theor. Appl. Genet. 113: 206–224.

Broman, K. W., and S. Sen, 2009 A Guide to QTL Mapping with R/qtl, Springer, New York.

Broman, K. W., H. Wu, S. Sen, and G. A. Churchill, 2003 R/qtl: QTL mapping in experimental crosses. Bioinformatics 19: 889–890.

Hyten, D. L., I.-Y. Choi, Q. Song, J. E. Specht, T. E. Carter et al., 2010 A high density integrated genetic linkage map of soybean and the development of a 1536 Universal Soy Linkage Panel for quantitative trait locus mapping. Crop Sci. 50: 960–968.

Lincoln, S. E., and E. S. Lander, 1992 Systematic detection of errors in genetic linkage data. Genomics 14: 604–610.

Manichaikul, A., A. A. Abraham, S. Sen, and K. W. Broman,2007 Significance thresholds for quantitative trait mapping under selective genotyping. Genetics 177: 1963–1966.

Martinez, O., 1996 Spurious linkage between markers in QTL mapping. Theor. Appl. Genet. 85: 480–488.

Saghai-Maroof, M. A., K. M. Soliman, R. A. Jorgensen, and R. W. Allard, 1984 Ribosomal DNA spacer-length polymorphisms in barley: Mendelian inheritance, chromosomal location, and population dynamics. Proc. Natl. Acad. Sci. USA 81: 8014–8018.

Zhang, L., S. Wang, H. Li, Q. Deng, A. Zheng et al., 2010 Effects of missing marker and segregation distortion on QTL mapping in F2 populations. Theor. Appl. Genet. 121: 1071–1082.
