# Supplementary material for: Multi-Population Selective Genotyping to Identify Soybean [Glycine max (L.) Merr.] Seed Protein and Oil QTLs
Source: G3 (Bethesda). 2016 Apr 1;6(6):1635–48. doi: 10.1534/g3.116.027656 (PMC4889660; doi:10.1534/g3.116.027656)
Supplement: Supplemental Material [file supp_g3.116.027656_FigureS1.pdf]

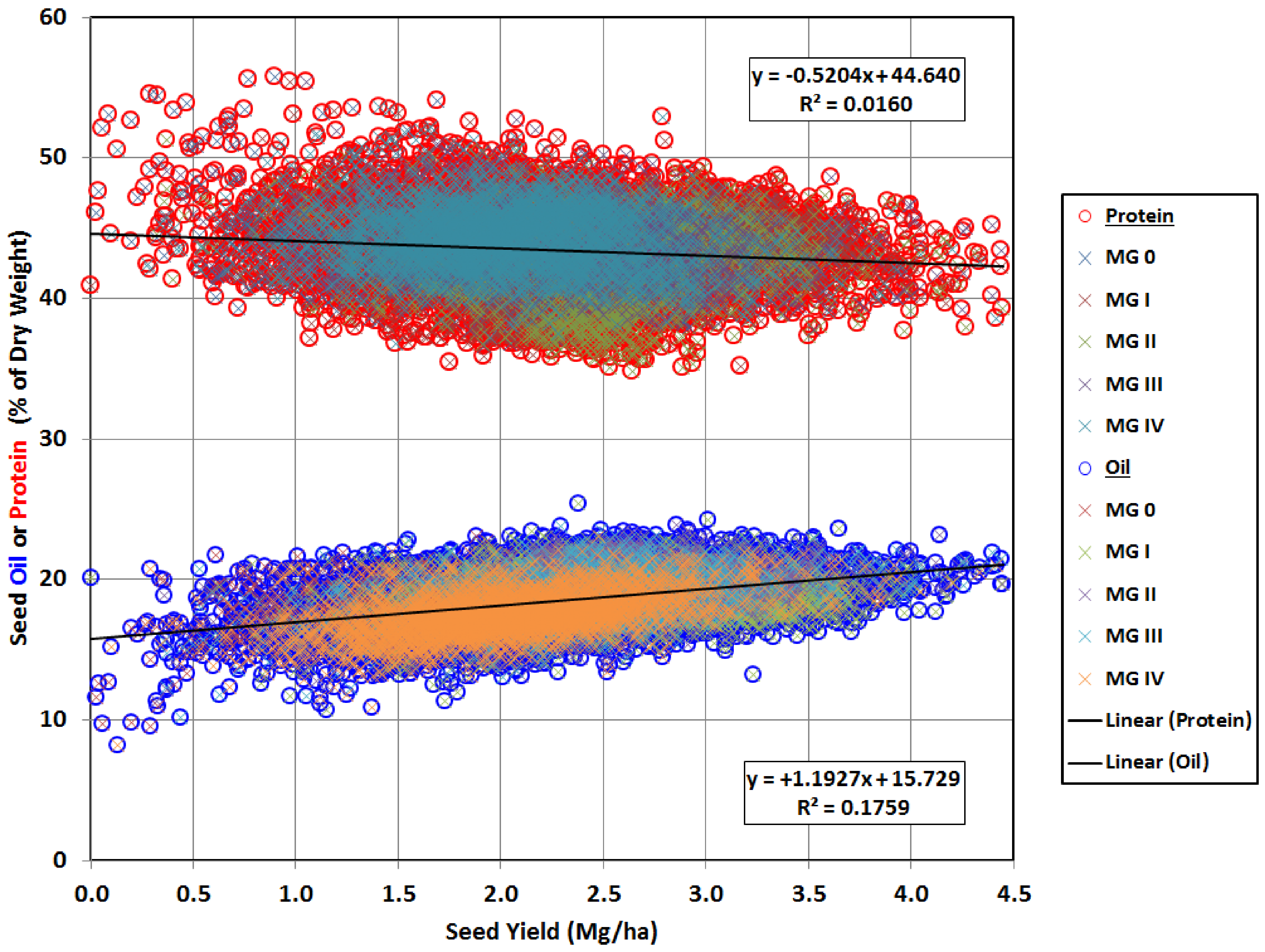

60
50
40
30
20
10
0
Seed Oil or Protein (% of Dry Weight)
0.0
0.5
1.0
1.5
2.0
2.5
3.0
3.5
4.0
4.5
Seed Yield (Mg/ha)
y = -0.5204x + 44.640
R² = 0.0160
y = +1.1927x + 15.729
R² = 0.1759
Protein
MG 0
MG I
MG II
MG III
MG IV
Oil
MG 0
MG I
MG II
MG III
MG IV
Linear (Protein)
Linear (Oil)

FIGURE S1. Soybean seed protein and oil values plotted against the corresponding seed yield values for maturity group (MG) 0 to IV accessions in the [*Glycine. max* (L.) Merr.] germplasm collection. Only 11,473 of the 12,141 accessions in these MGs have in-common values for all three traits. These data were provided courtesy of the soybean germplasm curator (R.L. Nelson, USDA-ARS, Urbana, IL).
