# Supplementary material for: Multi-Population Selective Genotyping to Identify Soybean [Glycine max (L.) Merr.] Seed Protein and Oil QTLs
Source: G3 (Bethesda). 2016 Apr 1;6(6):1635–48. doi: 10.1534/g3.116.027656 (PMC4889660; doi:10.1534/g3.116.027656)
Supplement: Supplemental Material [file supp_g3.116.027656_FigureS2.pdf]

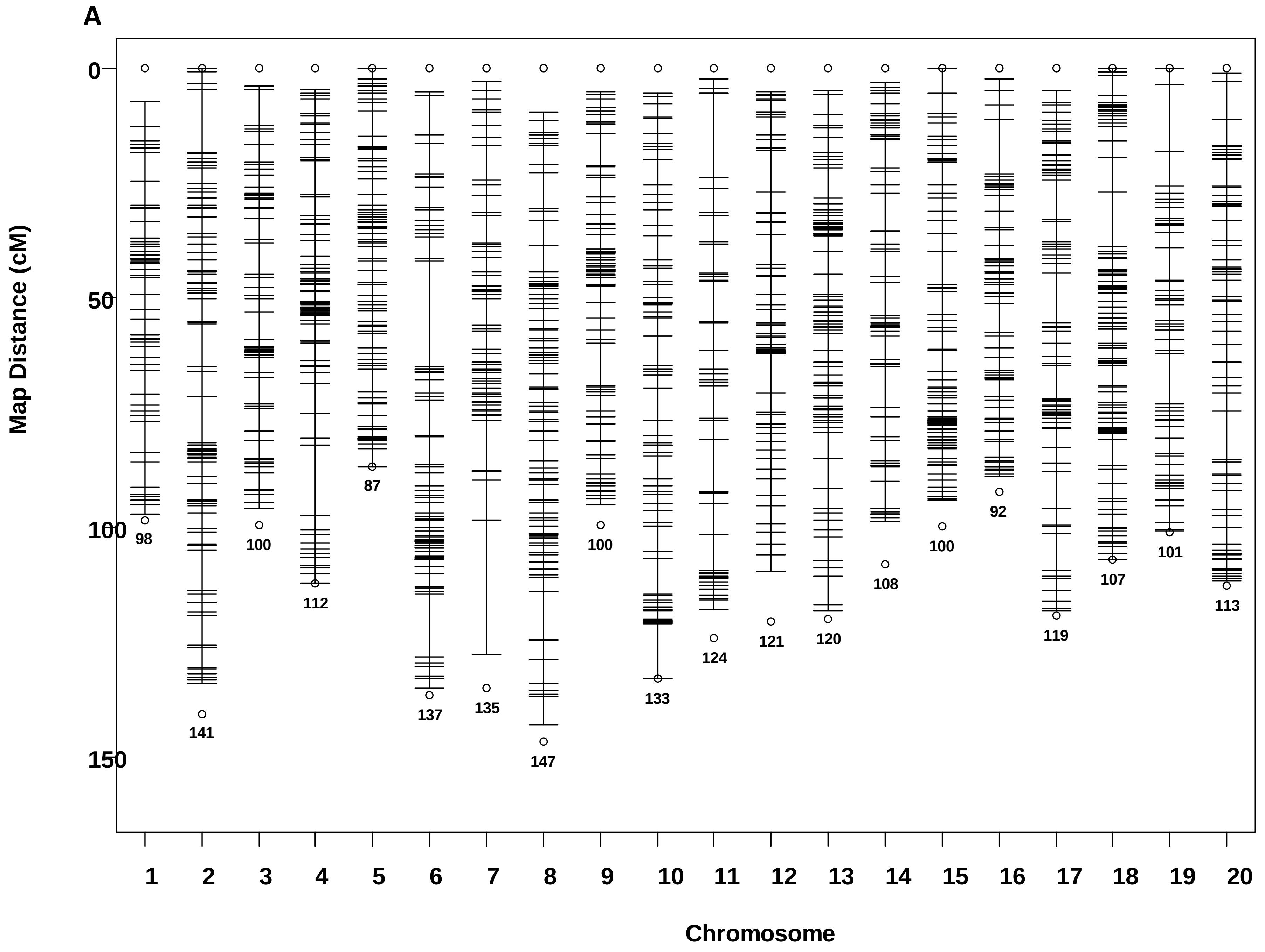
A
Map Distance (cM)
0
50
100
150
98
141
100
112
87
137
135
147
100
133
124
121
120
108
100
92
119
107
101
113
1
2
3
4
5
6
7
8
9
10
11
12
13
14
15
16
17
18
19
20
Chromosome

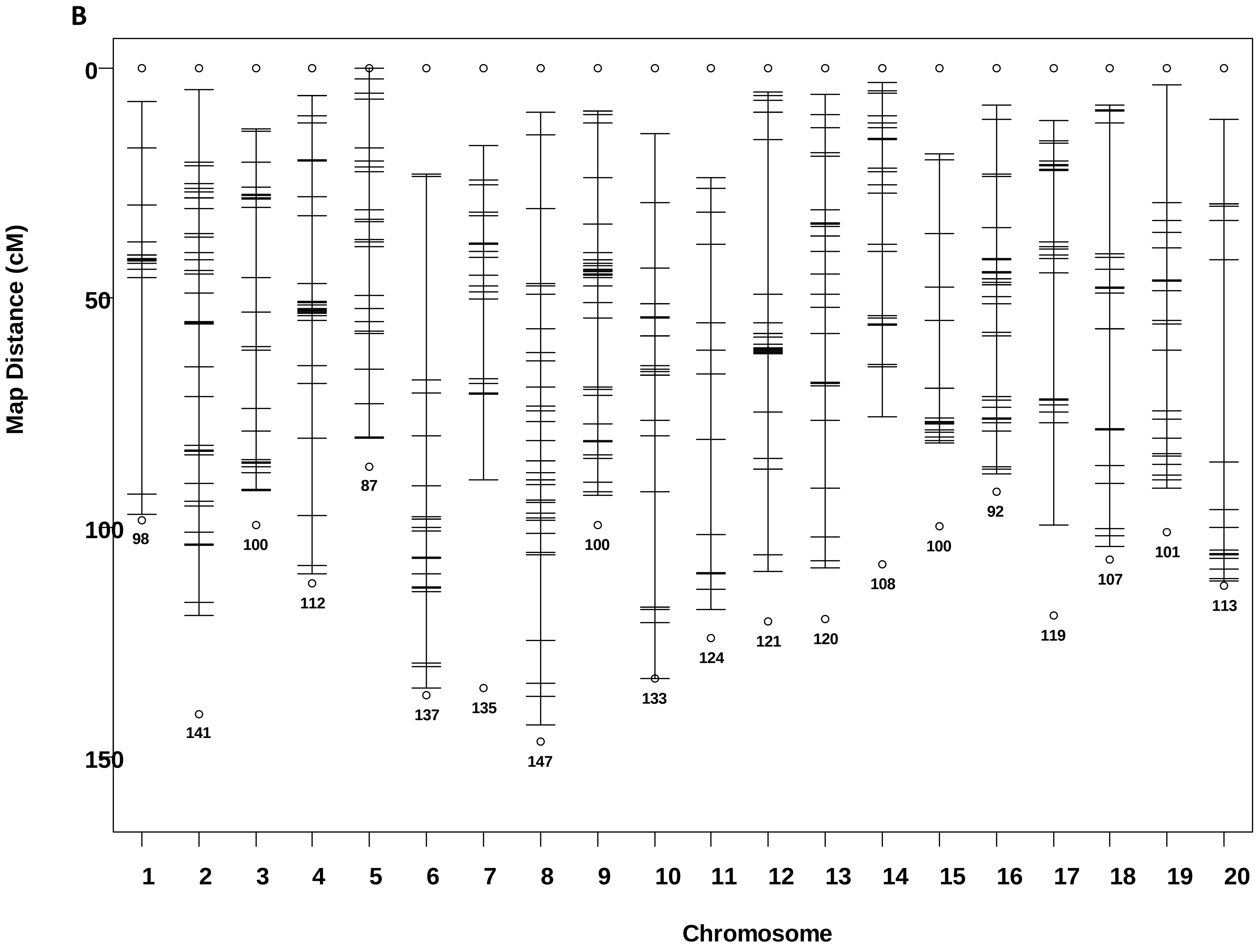
B
Map Distance (cM)
0
50
100
150
98
141
100
112
87
137
135
147
100
133
124
121
120
108
100
92
119
107
101
113
1
2
3
4
5
6
7
8
9
10
11
12
13
14
15
16
17
18
19
20
Chromosome

**FIGURE S2.** SNP marker map position (cross-hairs) in the 20 soybean chromosomes for (A) all of the 1536 SNPs in the chip developed by Hyten *et al.* (2010) and (B) just the 452 SNPs segregating in (the example) mating 1 of the 48 $F_2$ populations examined in this study. Open circles denote proximal and distal ends of each chromosome in the Version 4.0 genetic map (5500 markers of all types) that has a total genomic (Kosambi) distance of 2296.4 cM, but totals to only 2156.2 cM for a map that includes only the 1536 SNP markers.
