# Supplementary material for: Multi-Population Selective Genotyping to Identify Soybean [Glycine max (L.) Merr.] Seed Protein and Oil QTLs
Source: G3 (Bethesda). 2016 Apr 1;6(6):1635–48. doi: 10.1534/g3.116.027656 (PMC4889660; doi:10.1534/g3.116.027656)
Supplement: Supplemental Material [file supp_g3.116.027656_FigureS3.pdf]

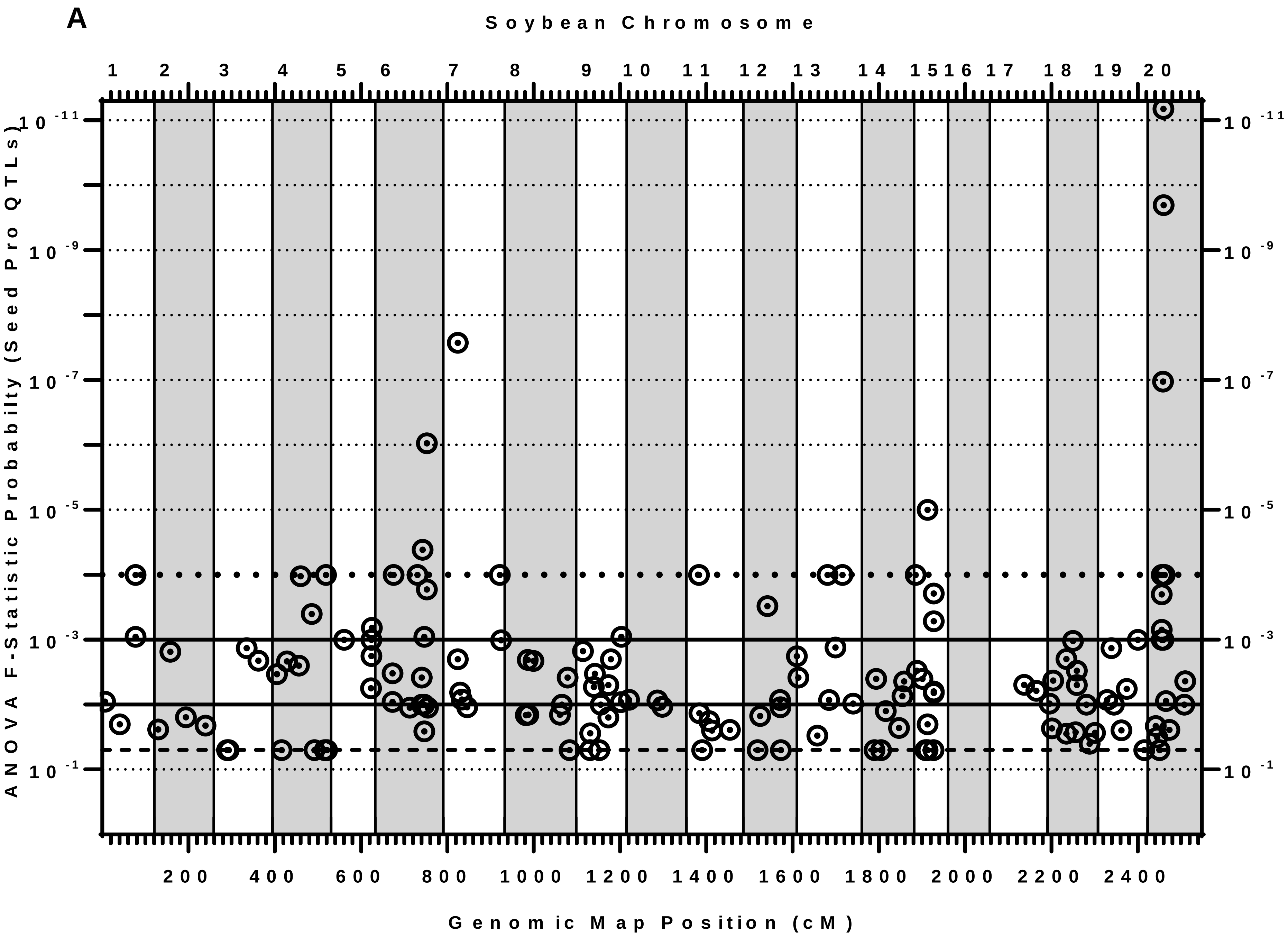
A
Soybean Chromosome
1 2 3 4 5 6 7 8 9 10 11 12 13 14 15 16 17 18 19 20
ANOVA F-Statistic Probabilty (Seed Pro QTLs)
10^{-11}
10^{-9}
10^{-7}
10^{-5}
10^{-3}
10^{-1}
200 400 600 800 1000 1200 1400 1600 1800 2000 2200 2400
Genomic Map Position (cM)

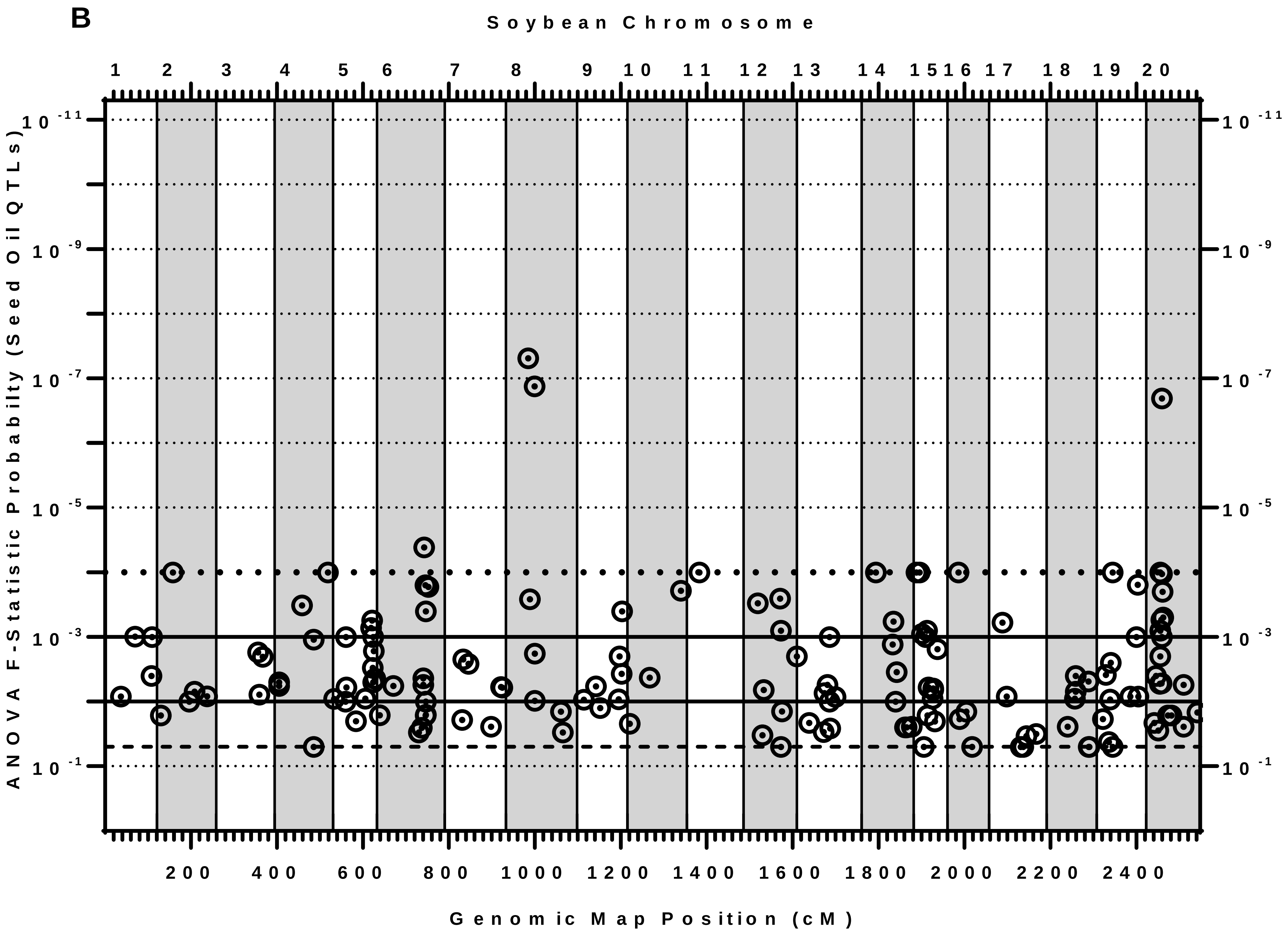
B
Soybean Chromosome
1 2 3 4 5 6 7 8 9 10 11 12 13 14 15 16 17 18 19 20
10 -11
10 -9
10 -7
10 -5
10 -3
10 -1
ANOVA F-Statistic Probabilty (Seed Oil QTLs)
200 400 600 800 1000 1200 1400 1600 1800 2000 2200 2400
Genomic Map Position (cM)

FIGURE S3. A graph of ANOVA F-statistic probabilities for (A) seed protein QTLs and (B) seed oil QTLs listed in SoyBase (Grant *et al.* 2010). SoyBase provides only Version 3.0 genetic map positions. ANOVA-based QTL detection was used in older reports, so for a common-unit comparative purpose, the LOD scores values provided in more recent reports were converted to F-statistic p-values. Because of the comparison-wise nature of F-statistic p-values, horizontal lines were placed at x-axis 10^{-x} values equivalent to p-values of 0.05 (dashed), 0.01 and 0.001 (solid), and 0.0001 (dotted) for reader convenience when comparing this graph with Figure 3 graph.
