# Supplementary material for: Multi-Population Selective Genotyping to Identify Soybean [Glycine max (L.) Merr.] Seed Protein and Oil QTLs
Source: G3 (Bethesda). 2016 Apr 1;6(6):1635–48. doi: 10.1534/g3.116.027656 (PMC4889660; doi:10.1534/g3.116.027656)
Supplement: Supplemental Material [file supp_g3.116.027656_TableS2.pdf]

**TABLE S2.** $F_1$ , $F_2$, $F_{1:2}$ and $F_{2:3}$ plants, seeds, and progeny numbers in each of the 48 matings, ordered by MG then by mating code and ID number. Population distributional statistics are shown for the one replicate seed protein (and oil) assay (all progeny) in each mating. Only those progeny in the lowest and highest quintiles of the protein distribution, based on quintile cut-off values, were selected for a two replicate seed protein assay. Using the 2-replicate means, progenies with the 22 lowest and 22 highest seed protein were identified, and their respective 44 $F_2$ progenitors were selectively genotyped (SG). The final percentage of $F_2$ plants chosen for selective genotyping is shown in the last column.

| | | | Female | | | | | One-Replicate Seed Protein | | | | | Seed Oil | | Protein-Oil | Two-Replicate Mean Seed Protein Value | | | | | | $F_2$ plants |
|---|---|---|---|---|---|---|---|---|---|---|---|---|---|---|---|---|---|---|---|---|---|---|
| Mating | | | Parent | $F_1$ | $F_{1:2}$ | $F_2$ | $F_{2:3}$ | $F_{2:3}$ Distributional Statistics | | | | | Statistics | | Phenotype | Low Pro 22 | | Quintile Cut | | High Pro 22 | | Selectively |
| No. | ID | MG | Accession | Plts | Sds | Plts | Prog | Min | MN | Max | Var | $H^2$ | Var | $H^2$ | Correlation | Min | Max | Low | High | Min | Max | Genotyped |
| | | | | ---------- number ---------- | | | | ------- g $kg^{-1}$ ------- | | | | | | | | --------------------- g kg-1 --------------------- | | | | | | - % - |
| 1 | 1001 | 000 | PI 153296 | 17 | 397 | 265 | 253 | 388 | 432 | 483 | 32 | 72 | 36 | 70 | -0.85 | 399 | 414 | 437 | 448 | 465 | 488 | 17.4 |
| 2 | 1002 | 000 | PI 189963 | 19 | 666 | 277 | 269 | 396 | 435 | 487 | 29 | 74 | 34 | 80 | -0.86 | 398 | 418 | 437 | 442 | 467 | 491 | 16.4 |
| 3 | 1003 | 000 | PI 548399 | 9 | 394 | 260 | 251 | 398 | 437 | 484 | 29 | 74 | 37 | 82 | -0.85 | 400 | 423 | 440 | 441 | 466 | 489 | 17.5 |
| 4 | 1004 | 000 | PI 372423 | 19 | 659 | 254 | 251 | 400 | 439 | 494 | 30 | 75 | 33 | 80 | -0.85 | 401 | 422 | 438 | 446 | 468 | 506 | 17.5 |
| 5 | 1005 | 000 | FC 30687 | 19 | 649 | 261 | 255 | 389 | 433 | 483 | 36 | 79 | 38 | 82 | -0.88 | 392 | 411 | 432 | 440 | 466 | 483 | 17.3 |
| 6 | 1006 | 000 | PI 153293 | 8 | 311 | 267 | 260 | 385 | 438 | 497 | 36 | 79 | 39 | 83 | -0.88 | 395 | 416 | 443 | 437 | 469 | 505 | 16.9 |
| 7 | 1007 | 000 | PI 372412 | 13 | 389 | 269 | 261 | 401 | 437 | 479 | 28 | 73 | 35 | 81 | -0.87 | 403 | 419 | 434 | 441 | 465 | 483 | 16.9 |
| 8 | 1009 | 000 | PI 548414 | 17 | 537 | 270 | 268 | 393 | 437 | 489 | 28 | 73 | 36 | 81 | -0.88 | 402 | 418 | 436 | 444 | 466 | 486 | 16.4 |
| 9 | 1022 | 00 | PI 153302 | 10 | 332 | 265 | 254 | 391 | 422 | 462 | 20 | 82 | 24 | 85 | -0.76 | 395 | 405 | 419 | 419 | 446 | 462 | 17.3 |
| 10 | 1023 | 00 | PI 159764 | 17 | 358 | 252 | 242 | 397 | 430 | 483 | 31 | 86 | 29 | 88 | -0.82 | 399 | 415 | 436 | 432 | 456 | 481 | 18.2 |
| 11 | 1024 | 00 | PI 438415 | 16 | 345 | 264 | 259 | 388 | 425 | 457 | 19 | 81 | 22 | 84 | -0.79 | 399 | 408 | 430 | 419 | 445 | 458 | 17.0 |
| 12 | 1025 | 00 | PI 153301 | 18 | 287 | 265 | 258 | 395 | 423 | 463 | 19 | 80 | 26 | 86 | -0.79 | 395 | 408 | 428 | 433 | 448 | 467 | 17.1 |
| 13 | 1026 | 00 | PI 189880 | 18 | 184 | 178 | 173 | 395 | 419 | 450 | 14 | 74 | 12 | 72 | -0.78 | 398 | 408 | 418 | 427 | 435 | 451 | 25.4 |
| 14 | 1027 | 00 | PI 153297 | 16 | 431 | 257 | 250 | 395 | 428 | 481 | 21 | 82 | 31 | 89 | -0.78 | 400 | 411 | 422 | 423 | 448 | 480 | 17.6 |
| 15 | 2211 | 00 | HHP | 8 | 524 | 219 | 147 | 383 | 434 | 482 | 53 | 87 | 57 | 93 | -0.81 | 387 | 417 | 430 | 450 | 456 | 480 | 29.9 |
| 16 | 2212 | 00 | AC Proteus | 9 | 405 | 286 | 278 | 392 | 418 | 450 | 13 | 45 | 15 | 60 | -0.73 | 393 | 407 | 424 | 414 | 432 | 451 | 15.8 |
| 17 | 2213 | 00 | AC Proteina | 16 | 466 | 288 | 275 | 387 | 416 | 446 | 17 | 59 | 20 | 70 | -0.80 | 390 | 403 | 434 | 414 | 441 | 458 | 16.0 |
| 18 | 1039 | 0 | PI 427138 | 20 | 761 | 266 | 257 | 385 | 419 | 464 | 17 | 61 | 28 | 83 | -0.75 | 390 | 405 | 424 | 429 | 441 | 462 | 17.1 |
| 19 | 1040 | 0 | PI 261469 | 15 | 691 | 260 | 238 | 375 | 422 | 463 | 23 | 71 | 32 | 85 | -0.80 | 380 | 407 | 422 | 426 | 448 | 463 | 18.5 |
| 20 | 1041 | 0 | PI 181571 | 24 | 710 | 268 | 248 | 388 | 419 | 454 | 18 | 62 | 30 | 84 | -0.71 | 394 | 407 | 422 | 424 | 443 | 460 | 17.7 |
| 21 | 1042 | 0 | PI 424148 | 8 | 297 | 219 | 210 | 392 | 424 | 465 | 21 | 68 | 30 | 84 | -0.76 | 396 | 412 | 427 | 419 | 447 | 462 | 21.0 |
| 22 | 1043 | 0 | PI 423954 | 13 | 379 | 267 | 248 | 383 | 429 | 464 | 26 | 73 | 27 | 82 | -0.73 | 363 | 408 | 428 | 428 | 445 | 461 | 17.7 |
| 23 | 1044 | 0 | PI 154196 | 18 | 1117 | 271 | 245 | 384 | 419 | 454 | 21 | 67 | 28 | 83 | -0.78 | 385 | 405 | 428 | 424 | 443 | 461 | 18.0 |

| | | | | | | | | | | | | | | | | | | | | | | |
|---|---|---|---|---|---|---|---|---|---|---|---|---|---|---|---|---|---|---|---|---|---|---|
| 24 | 1054 | I | PI 437088A | 7 | 789 | 225 | 184 | 402 | 435 | 481 | 24 | 70 | 26 | 20 | -0.80 | 406 | 423 | 432 | 429 | 454 | 487 | 23.9 |
| 25 | 1055 | I | PI 423949 | 12 | 1014 | 286 | 265 | 386 | 428 | 476 | 24 | 69 | 42 | 50 | -0.82 | 392 | 410 | 422 | 423 | 448 | 477 | 16.6 |
| 26 | 1056 | I | PI 427141 | 11 | 906 | 285 | 258 | 392 | 431 | 471 | 27 | 73 | 39 | 46 | -0.77 | 397 | 411 | 427 | 431 | 458 | 477 | 17.1 |
| 27 | 1057 | I | PI 437716A | 6 | 497 | 272 | 246 | 392 | 425 | 460 | 17 | 58 | 27 | 21 | -0.67 | 396 | 413 | 437 | 433 | 446 | 468 | 17.9 |
| 28 | 1058 | I | PI 423942 | 6 | 548 | 279 | 246 | 379 | 432 | 480 | 27 | 73 | 42 | 50 | -0.76 | 375 | 411 | 431 | 428 | 455 | 482 | 17.9 |
| 29 | 1075 | II | PI 423948A | 2 | 238 | 208 | 183 | 372 | 430 | 475 | 38 | 72 | 43 | 66 | -0.75 | 370 | 408 | 423 | 439 | 454 | 474 | 24.0 |
| 30 | 1076 | II | PI437112A | 11 | 703 | 217 | 188 | 371 | 431 | 483 | 34 | 69 | 43 | 66 | -0.76 | 375 | 410 | 425 | 439 | 451 | 479 | 23.4 |
| 31 | 1098 | II | PI 548608 | 14 | 685 | 254 | 230 | 389 | 419 | 457 | 15 | 31 | 21 | 29 | -0.66 | 389 | 405 | 419 | 427 | 435 | 456 | 19.1 |
| 32 | 1107 | III | PI 445845 | 14 | 891 | 278 | 222 | 384 | 426 | 471 | 31 | 57 | 36 | 49 | -0.78 | 385 | 407 | 433 | 421 | 450 | 473 | 19.8 |
| 33 | 1108 | III | PI 398516 | 12 | 966 | 280 | 226 | 376 | 423 | 466 | 27 | 50 | 29 | 37 | -0.74 | 374 | 403 | 419 | 432 | 443 | 464 | 19.5 |
| 34 | 1109 | III | PI 91725-4 | 15 | 838 | 263 | 225 | 383 | 425 | 480 | 30 | 55 | 50 | 64 | -0.76 | 387 | 404 | 426 | 439 | 445 | 486 | 19.6 |
| 35 | 1110 | III | PI 340011 | 13 | 833 | 254 | 208 | 384 | 425 | 476 | 30 | 55 | 36 | 49 | -0.81 | 385 | 405 | 421 | 417 | 444 | 474 | 21.2 |
| 36 | 1111 | III | PI 243532 | 9 | 489 | 273 | 216 | 386 | 425 | 496 | 42 | 68 | 52 | 65 | -0.74 | 386 | 405 | 429 | 440 | 449 | 497 | 20.4 |
| 37 | 1113 | III | PI 408138C | 12 | 1042 | 259 | 219 | 391 | 430 | 495 | 33 | 59 | 31 | 41 | -0.78 | 390 | 409 | 429 | 428 | 450 | 498 | 20.1 |
| 38 | 1121 | III | PI 398672 | 14 | 670 | 262 | 217 | 377 | 419 | 464 | 31 | 56 | 31 | 42 | -0.73 | 380 | 400 | 425 | 429 | 443 | 472 | 20.3 |
| 39 | 1122 | III | PI 360843 | 18 | 1544 | 213 | 189 | 373 | 419 | 459 | 19 | 30 | 22 | 18 | -0.68 | 377 | 402 | 415 | 424 | 435 | 456 | 23.3 |
| 40 | 1138 | IV | PI 253666A | 9 | 735 | 242 | 141 | 376 | 431 | 494 | 42 | 76 | 58 | 73 | -0.80 | 379 | 413 | 434 | 446 | 453 | 493 | 31.2 |
| 41 | 1139 | IV | PI 407788A | 17 | 1399 | 283 | 167 | 391 | 434 | 476 | 32 | 69 | 54 | 71 | -0.83 | 391 | 412 | 420 | 447 | 455 | 484 | 26.3 |
| 42 | 1140 | IV | PI 424286 | 16 | 554 | 264 | 189 | 389 | 429 | 467 | 27 | 63 | 39 | 60 | -0.83 | 389 | 411 | 431 | 441 | 448 | 465 | 23.3 |
| 43 | 1142 | IV | PI 407877B | 10 | 936 | 278 | 217 | 397 | 429 | 473 | 21 | 52 | 33 | 54 | -0.80 | 397 | 412 | 423 | 425 | 447 | 482 | 20.3 |
| 44 | 1143 | IV | PI 398704 | 5 | 415 | 279 | 195 | 378 | 431 | 471 | 32 | 69 | 54 | 71 | -0.83 | 380 | 411 | 422 | 446 | 455 | 475 | 22.6 |
| 45 | 1145 | IV | PI 398970 | 1 | 85 | 165 | 115 | 381 | 428 | 478 | 38 | 74 | 48 | 67 | -0.81 | 384 | 413 | 416 | 438 | 442 | 473 | 38.3 |
| 46 | 1146 | IV | PI 407823 | 5 | 2190 | 254 | 171 | 397 | 424 | 467 | 19 | 48 | 40 | 62 | -0.75 | 396 | 408 | 416 | 432 | 440 | 463 | 25.7 |
| 47 | 1152 | IV | PI 407773B | 2 | 500 | 260 | 145 | 394 | 425 | 465 | 26 | 61 | 40 | 62 | -0.81 | 393 | 410 | 416 | 420 | 439 | 463 | 30.3 |
| 48 | 1183 | V | PI 458256 | 9 | 870 | 288 | 231 | 377 | 411 | 451 | 15 | 34 | 15 | 0 | -0.68 | 376 | 396 | 417 | 405 | 425 | 448 | 19.0 |
