# Supplementary material for: Multi-Population Selective Genotyping to Identify Soybean [Glycine max (L.) Merr.] Seed Protein and Oil QTLs
Source: G3 (Bethesda). 2016 Apr 1;6(6):1635–48. doi: 10.1534/g3.116.027656 (PMC4889660; doi:10.1534/g3.116.027656)
Supplement: Supplemental Material [file supp_g3.116.027656_TableS3.pdf]

TABLE S3. The number of SNP markers detected as segregating in each soybean chromosome in each of the 48 F_2 populations *versus* the 1536 potentially detectable SNPs on the USLP 1.0 chip (Hyten *et al.* 2010). Some selectively genotyped F_2 individuals and some SNP markers were removed from the populations during the R/qtl error-checking phase of the project. Thus, final F_2 phenotype (Phe) and genotype (Gen) numbers, final selective genotyping (SG) percentage, final total non-missing genotype percentage, and final F_2 AA:AB:BB segregation ratios (summed over SNPs) are presented here for each mating. Relative to the last three F_2 SNP segregation columns, the high protein parent was arbitrarily assigned the BB genotype code at each SNP locus.

							Soybean Chromosome:																				All		SNP Marker		
							1	2	3	4	5	6	7	8	9	10	11	12	13	14	15	16	17	18	19	20	Chr		Segregation		
Mating			Final F_2 Data			Non	---- Number of SNP markers on the Soybean USLP 1.0 Chip ----																					Pop	Ratio (%)		
No.	ID	MG	Phe	Gen	SG	Miss	63	90	67	81	80	92	65	96	84	72	54	63	92	61	85	68	78	115	56	74	1536	SNP	AA	AB	BB
			---- no. ----		%	%	---- Number of SNP markers detected as parentally polymorphic ----																					%	---- % ----		
1	1001	000	253	44	17.4	16.1	15	34	24	26	23	20	18	33	32	22	13	24	25	21	16	29	20	20	20	17	452	29.4	23.5	50.9	25.5
2	1002	000	266	41	15.4	13.8	13	32	24	24	23	17	17	33	30	21	13	22	27	20	14	29	20	15	20	16	430	28.0	21.0	58.1	21.0
3	1003	000	251	44	17.5	16.0	13	29	16	21	21	20	16	23	28	18	13	21	20	13	13	23	19	18	17	12	374	24.3	23.7	51.7	24.6
4	1004	000	247	40	16.2	14.5	13	29	18	21	23	20	18	23	27	18	12	21	23	13	13	24	19	16	16	13	380	24.7	20.1	55.9	24.0
5	1005	000	254	43	16.9	16.5	18	35	26	28	26	22	19	37	33	23	16	22	28	24	17	29	23	21	24	17	488	31.8	24.6	48.2	27.2
6	1006	000	260	44	16.9	16.0	18	35	26	27	27	22	19	35	31	23	16	22	27	24	17	29	22	21	24	18	483	31.4	21.5	52.4	26.1
7	1007	000	261	44	16.9	15.5	14	23	13	13	19	13	8	27	24	14	10	15	19	16	12	22	14	17	11	13	317	20.6	25.1	50.8	24.1
8	1009	000	266	42	15.8	14.3	14	22	11	11	19	13	7	26	23	13	9	14	18	16	12	21	14	17	11	14	305	19.9	19.6	58.3	22.1
9	1022	00	248	38	15.3	14.1	26	34	27	33	28	21	12	34	21	18	17	19	30	18	30	30	23	26	20	16	483	31.4	22.6	51.9	25.5
10	1023	00	239	41	17.2	16.2	26	36	25	33	27	21	12	35	21	18	17	19	31	18	29	30	23	26	22	15	484	31.5	22.4	52.1	25.5
11	1024	00	257	42	16.3	15.8	26	36	27	33	28	21	12	32	21	18	17	19	31	18	29	30	23	24	22	16	483	31.4	23.6	52.9	23.5
12	1025	00	258	44	17.1	16.9	26	36	27	33	27	21	11	34	21	18	17	19	31	18	30	30	23	26	22	16	486	31.6	23.1	52.0	24.9
13	1026	00	173	44	25.4	24.6	19	33	27	20	21	22	16	28	22	9	21	18	36	18	30	20	20	19	17	15	431	28.1	24.1	52.0	23.9
14	1027	00	249	43	17.3	17.0	28	40	31	37	28	23	13	36	20	19	20	22	36	19	30	31	23	26	23	22	527	34.3	24.0	50.5	25.5
15	2211	00	147	44	29.9	29.5	10	42	19	26	30	26	10	16	20	19	20	20	22	9	29	17	16	46	9	19	425	27.7	25.1	47.6	27.3
16	2212	00	278	44	15.8	14.8	17	35	15	20	20	26	21	29	15	11	10	25	20	13	28	22	13	34	10	18	402	26.2	24.5	49.7	25.8
17	2213	00	269	38	14.1	13.8	17	41	20	24	27	24	18	20	16	13	12	22	29	15	27	22	13	23	10	19	412	26.8	27.2	49.0	23.8
18	1039	0	257	44	17.1	16.5	29	39	24	24	28	25	15	27	27	25	12	30	43	29	28	35	27	31	20	19	537	35.0	24.0	49.1	26.8
19	1040	0	237	43	18.1	17.6	29	39	24	23	28	25	19	26	29	24	13	30	43	27	28	35	27	33	20	19	541	35.2	26.3	49.2	24.6
20	1041	0	246	42	17.1	16.2	24	37	21	17	27	21	10	22	24	22	9	25	33	13	18	29	12	22	18	11	415	27.0	25.7	48.7	25.6
21	1042	0	208	42	20.2	19.4	29	38	28	20	23	24	14	27	27	23	15	28	41	29	31	32	20	30	20	16	515	33.5	22.9	50.4	26.6
22	1043	0	248	44	17.7	16.8	19	34	16	14	22	15	12	18	23	17	8	24	31	11	14	20	16	17	12	9	352	22.9	24.7	50.1	25.2
23	1044	0	241	40	16.6	15.7	22	29	27	28	17	20	16	18	27	16	18	20	35	23	31	35	15	35	15	17	464	30.2	24.0	50.5	25.5

24	1054	I	184	44	23.9	22.3	16	22	14	11	16	29	6	24	14	22	15	17	20	14	25	15	13	24	16	15	348	22.7	23.3	52.5	24.7
25	1055	I	264	43	16.3	15.6	20	28	22	33	20	22	13	24	20	26	17	27	27	21	28	25	20	21	18	18	450	29.3	24.0	52.4	23.6
26	1056	I	257	43	16.7	16.1	21	23	20	29	26	25	14	20	22	24	14	27	27	20	22	22	22	23	15	16	432	28.1	27.3	50.1	22.6
27	1057	I	246	44	17.9	17.1	12	27	19	19	27	18	13	14	27	17	16	11	20	13	26	26	17	30	9	18	379	24.7	24.2	52.5	23.3
28	1058	I	243	41	16.9	16.4	26	23	15	25	20	20	10	22	22	25	18	26	30	26	23	30	19	24	20	20	444	28.9	25.4	48.9	25.7
29	1075	II	181	42	23.2	22.7	24	23	15	21	28	25	14	19	20	18	14	23	19	18	28	28	12	32	21	17	419	27.3	25.3	49.6	25.1
30	1076	II	187	43	23.0	22.6	24	21	25	23	27	43	37	18	34	27	18	24	28	18	33	29	21	34	28	20	532	34.6	24.1	50.9	25.0
31	1098	II	229	43	18.8	18.0	11	19	26	7	18	6	3	5	10	17	7	8	12	13	16	12	16	26	8	19	259	16.9	30.4	46.0	23.6
32	1107	III	221	43	19.5	19.1	20	31	26	26	38	18	19	30	21	21	17	26	32	24	26	23	20	45	16	30	509	33.1	29.6	48.3	22.1
33	1108	III	226	44	19.5	19.1	25	19	22	24	24	27	16	23	20	17	24	28	35	19	35	28	18	42	20	12	478	31.1	25.7	51.2	23.1
34	1109	III	225	44	19.6	19.0	19	37	18	32	29	26	11	28	19	20	17	25	37	19	27	24	23	40	20	24	495	32.2	23.8	50.2	25.9
35	1110	III	208	44	21.2	21.0	18	40	21	27	38	17	22	36	17	25	20	27	36	22	30	27	21	41	18	30	533	34.7	25.8	49.5	24.7
36	1111	III	214	42	19.6	18.3	25	40	20	21	34	22	18	23	18	16	20	24	26	13	23	28	25	29	22	24	471	30.7	24.4	50.2	25.4
37	1113	III	219	44	20.1	19.9	18	37	19	28	28	13	18	34	16	17	18	24	37	28	26	30	20	45	19	28	503	32.7	24.9	51.0	24.1
38	1121	III	217	44	20.3	19.5	18	26	18	22	32	19	12	28	12	26	21	24	21	21	25	34	20	44	20	27	470	30.6	25.2	49.5	25.3
39	1122	III	189	44	23.3	22.5	25	25	15	16	20	14	15	24	19	22	14	14	21	13	24	29	17	26	20	24	397	25.8	23.3	48.7	28.0
40	1138	IV	141	44	31.2	30.7	28	34	19	20	34	31	23	38	38	21	24	28	24	13	40	19	31	40	18	37	560	36.5	26.0	49.9	24.1
41	1139	IV	166	43	25.9	24.7	17	41	24	23	30	22	22	25	24	19	12	20	34	16	21	19	26	47	19	25	486	31.6	24.3	47.1	28.6
42	1140	IV	188	43	22.9	22.8	14	33	24	20	26	30	16	33	27	24	18	30	24	8	22	23	25	39	23	25	484	31.5	25.5	47.3	27.1
43	1142	IV	217	44	20.3	20.0	16	42	23	19	23	30	20	24	16	19	12	26	40	14	29	27	25	46	18	30	499	32.5	25.9	49.8	24.3
44	1143	IV	194	43	22.2	21.3	12	38	22	18	24	28	22	30	20	17	21	23	28	12	25	24	25	46	28	36	499	32.5	25.3	48.2	26.5
45	1145	IV	115	44	38.3	35.8	10	43	20	19	28	22	22	26	21	15	18	25	32	9	19	20	19	33	19	27	447	29.1	24.0	53.8	22.3
46	1146	IV	165	38	23.0	22.1	17	28	17	22	22	24	23	20	13	14	13	20	24	14	21	20	13	36	21	14	396	25.8	26.5	49.8	23.7
47	1152	IV	144	43	29.9	29.7	15	44	15	19	15	30	23	17	14	22	17	23	17	12	23	24	19	39	24	16	428	27.9	24.1	49.4	26.5
48	1183	V	230	43	18.7	18.6	14	44	26	17	26	27	13	25	19	24	21	31	33	10	24	30	30	39	25	34	512	33.3	24.1	49.2	26.7
